# Analytical profile of *N*-ethyl-*N*-cyclopropyl lysergamide (ECPLA), an isomer of lysergic acid 2,4-dimethylazetidide (LSZ)

**DOI:** 10.1002/dta.2911

**Published:** 2020-09-17

**Authors:** Simon D. Brandt, Pierce V. Kavanagh, Folker Westphal, Alexander Stratford, Simon P. Elliott, Geraldine Dowling, Adam L. Halberstadt

**Affiliations:** 1 School of Pharmacy and Biomolecular Sciences, Liverpool John Moores University, Liverpool, UK; 2 Department of Pharmacology and Therapeutics, School of Medicine, Trinity Centre for Health Sciences, St James Hospital, Dublin, Ireland; 3 State Bureau of Criminal Investigation Schleswig-Holstein, Section Narcotics/Toxicology, Kiel, Germany; 4 Synex Synthetics BV, Maastricht, The Netherlands; 5 Elliott Forensic Consulting, Birmingham, UK; 6 Department of Life Sciences, School of Science, Sligo Institute of Technology, Sligo, Ireland; 7 Department of Psychiatry, University of California San Diego, La Jolla, CA, USA; 8 Research Service, VA San Diego Healthcare System, La Jolla, CA, USA

**Keywords:** forensic, isomers, LSD, new psychoactive substances, psychedelics

## Abstract

Recent investigations have shown that *N*-ethyl-*N*-cyclopropyl lysergamide (ECPLA) produces LSD-like behavioral effects in mice, which suggests that it may act as a hallucinogen in humans. Although the use of ECPLA as a recreational drug has been limited, key analytical data that can be used to detect ECPLA are required for future forensic and clinical investigations. ECPLA is an isomer of (2′*S*,4′*S*)-lysergic acid 2,4-dimethylazetidide (LSZ), a lysergamide that emerged as a recreational drug in 2013. Several analytical approaches were examined, including single- and tandem mass spectrometry platforms at low and high resolution, gas- and liquid chromatography (GC, LC), nuclear magnetic resonance spectroscopy (NMR), and GC condensed-phase infrared spectroscopy (GC-sIR). ECPLA and LSZ could be differentiated by NMR, GC-sIR, GC, and LC-based methods. The electron ionization mass spectra of ECPLA and LSZ contained ion clusters typically observed with related lysergamides such as m/z 150–155, m/z 177–182, m/z 191–197, m/z 205–208, and m/z 219–224. One of the significant differences in abundance related to these clusters included ions at m/z 196 and m/z 207/208. The base peaks were detected at m/z 221 in both cases followed by the retro-Diels-Alder fragment at m/z 292. Minor but noticeable differences between the two isomers could also be seen in the relative abundance of m/z 98 and m/z 41. Electrospray ionization mass spectra included lysergamide-related ions at m/z 281, 251, 223, 208, 197, 180, and 140. LSZ (but not ECPLA) showed product ions at m/z 267 and m/z 98 under the conditions used.

## 1 ∣ INTRODUCTION

**FIGURE 1:**
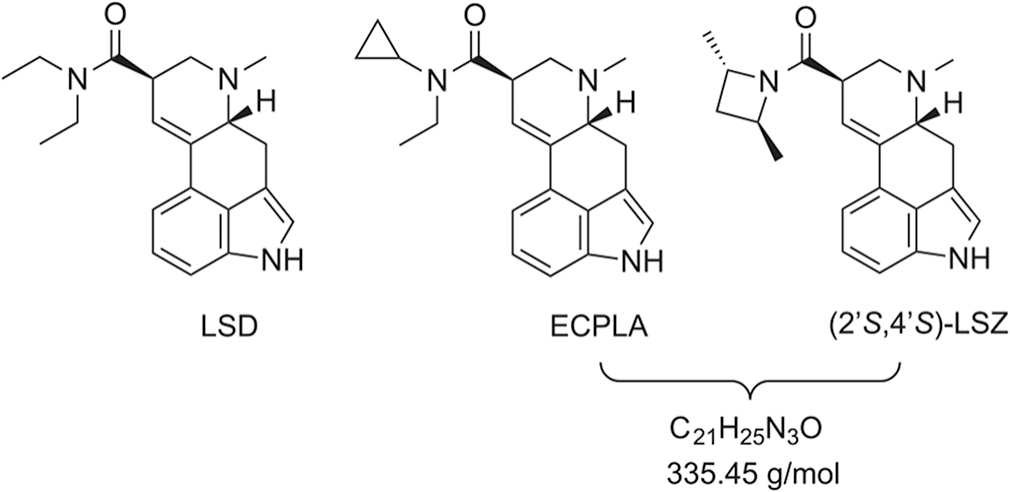
Chemical structures of LSD, *N*-ethyl-*N*-cyclopropyl lysergamide (ECPLA), and the isomeric (2′S,4′S)-lysergic acid 2,4-dimethylazetidide (LSZ)

Apart from being a popular recreational drug, lysergic acid diethylamide (LSD) (Figure 1) is also an important research tool for medical and scientific studies.^1-3^ Various modifications of the diethylamide moiety in LSD have been explored over the years, although most other *N*,*N*-dialkylamide substituents are associated with considerable reductions in potency and activity.^4-6^ However, the development of new lysergamides has helped to elucidate the structural features that govern their interactions with the 5-HT_2A_ receptor, which is believed to be the primary site of action in the brain for LSD and other hallucinogens.

*N*-Cyclopropyl-*N*-ethyl-6-methyl-9,10-didehydroergoline-8β-carboxamide (lysergic acid *N*-ethyl-*N*-cyclopropylamide, ECPLA) is one of the newest additions to the family of LSD analogs undergoing pharmacological evaluation (Figure 1). Similar to LSD, ECPLA has moderate to high affinity for most serotonin receptors, α_2_-adrenoceptor subtypes, and D_2_-like dopamine receptors. When evaluated in G_q_-mediated calcium flux assays, ECPLA acts as a potent, highly efficacious 5-HT_2A_ agonist. ECPLA was also found to induce the head-twitch response (a 5-HT_2A_-mediated behavior) in mice with an ED_50_ of 317.2 nmol/kg, which is about 40% of the potency of LSD.^6^ Taken together, these findings indicate that ECPLA has LSD-like psychopharmacology and likely acts as a hallucinogen in humans. Studies of the biotransformation of ECPLA under various in vitro conditions confirmed that it is metabolized through the same routes as LSD and other lysergamides.^7^

Although LSD has been a popular recreational drug since the 1960s, several other lysergamides have appeared in recent years. Most of those LSD analogs were known in the chemical and patent literature prior to their appearance as recreational drugs, but few appear to be true “designer drugs” with no existing scientific history.^8-13^ ECPLA is not currently available as a recreational drug and has so far not been identified in medical or legal investigations, but the dissemination of key analytical data for ECPLA is justified as a proactive step, given the popularity of other novel LSD analogs. The availability of analytical data for ECPLA will be useful for future forensic and clinical investigations. In addition, because the isomeric lysergamide (2′*S*,4′*S*)-lysergic acid 2,4-dimethylazetidide (LSZ, Figure 1) is a known recreational drug,^9^ it is necessary to identify analytical features that can be used to differentiate between ECPLA and LSZ.

## 2 ∣ EXPERIMENTAL

### 2.1 ∣ Materials

All chemicals used were analytical or HPLC grade and were obtained from Rathburn Chemicals Ltd (Walkerburn, Scotland, UK), Fisher Scientific (Dublin, Ireland), or Aldrich (Dorset, UK). *N*-Ethyl-*N*-cyclopropyl lysergamide (ECPLA) hemitartrate was available from previous studies.^6,7^

### 2.2 ∣ Instrumentation

#### 2.2.1 ∣ Gas chromatography–mass spectrometry (GC–MS)

The electron ionization (EI) mass spectrum was recorded using a Finnigan TSQ 7000 triple stage quadrupole mass spectrometer coupled to a gas chromatograph (Trace GC Ultra, Thermo Electron, Dreieich, Germany). Sample introduction was carried out using a CTC CombiPAL (CTC Analytics, Zwingen, Switzerland) autosampler. The emission current was 200 μA and the scan time was 1 s spanning a scan range of m/z 29–600. The ion source temperature was maintained at 175°C. Samples were introduced via gas chromatography (GC) with splitless injection using a fused silica capillary DB-1 column (30 m × 0.25 mm, film thickness 0.25 μm). The temperature program consisted of an initial temperature of 80°C, held for 1 min, followed by a ramp to 280°C at 15°C/min. The final temperature was held for 21 min and the injector temperature was 220°C. The transfer line temperature was maintained at 280°C and the carrier gas was helium in constant flow mode at a flow rate of 1.2 mL/min. Approximately 2 mg was dissolved in 1.5 mL chloroform. For analysis, 1 μL sample solution was injected into the GC–MS system. Retention indices (RI) are given as Kovats indices calculated from measurement of an *n*-alkane mixture analyzed with the above-mentioned temperature program.

#### 2.2.2 ∣ Gas chromatography-condensed-phase-infrared analysis (GC-sIR)

The ECPLA sample was analyzed using a GC-sIR system that consisted of an Agilent GC 7890B (Waldbronn, Germany) with a probe sampler Agilent G4567A and a DiscovIR-GC™ (Spectra Analysis, Marlborough, MA, USA). The column eluent was cryogenically accumulated on a spirally rotating ZnSe disk cooled by liquid nitrogen. IR spectra were recorded through the IR-transparent ZnSe disk using a nitrogen-cooled MCT detector. GC parameters: injection in splitless mode with an injection port temperature set at 240°C and a DB-1 fused silica capillary column (30 m × 0.32 mm i.d., 0.25 μm film thickness). The carrier gas was helium with a flow rate of 2.5 mL/min and the oven temperature program was as follows: 80°C for 2 min, ramped to 290°C at 20°C/min, and held at for 20 min. The transfer line was heated at 280°C. Infrared conditions: oven temperature, restrictor temperature, disc temperature, and Dewar cap temperatures were 280°C, 280°C, −40°C, and 35°C, respectively. The vacuum was 0.2 mTorr, disc speed 3 mm/s, spiral separation was 1 mm, wavelength resolution 4 cm^−1^ and IR range 650–4000 cm^−1^. The acquisition time was 0.6 s/file with 64 scans/spectrum. Data were processed using GRAMS/AI Ver. 9.1 (Grams Spectroscopy Software Suite, Thermo Fischer Scientific, Dreieich, Germany) followed by implementation of the OMNIC Software, Ver. 7.4.127 (Thermo Electron Corporation, Dreieich, Germany).

#### 2.2.3 ∣ Ultra-high performance liquid chromatography-electrospray ionization tandem mass spectrometry (UHPLC-QTOF-MS/MS)

UHPLC-QTOF-MS/MS data were obtained from an Agilent 6540 UHD Accurate-Mass Q-TOF LC-MS system coupled to an Agilent 1290 Infinity UHPLC system (Agilent, Cheshire, UK). Separation was achieved using an Agilent Zorbax Eclipse Plus C18 column (100 mm × 2.1 mm, 1.8 μm) (Agilent, Cheadle, UK). Mobile phases consisted of acetonitrile (containing 1% formic acid) and 1% formic acid in water. The column temperature was set at 40°C and data were acquired for 5.5 min. The flow rate was (0.6 mL/min). The gradient was set at 5–70% acetonitrile over 3.5 min, then increased to 95% acetonitrile in 1 min and held for 0.5 min before returning to 5% acetonitrile in 0.5 - min. QTOF-MS data were acquired in positive ion mode scanning from m/z 100–1000 with and without auto MS/MS fragmentation. Ionization was achieved with an Agilent JetStream electrospray source and infused internal reference masses. The ion source parameters were gas temperature 325°C, drying gas 10 L/min and sheath gas temperature 400°C. Internal reference ions at m/z 121.05087 and m/z 922.00979 were used for calibration purposes. The sample concentration was 10 μg/mL and dissolved in methanol.

#### 2.2.4 ∣ Liquid chromatography-electrospray ionization mass spectrometry (LC-Q-MS)

HPLC single quadrupole mass spectrometry (LC-Q-MS) analyses were performed on an Agilent 1100 system. Separation was obtained on a Restek (Bellefonte, PA, USA) Allure PFPP column (50 mm × 2.1 mm, 5 μm). Mobile phase A consisted of 0.1% formic acid in water, whereas mobile phase B consisted of 0.1% formic acid in acetonitrile. The Agilent 1100 LC-MSD settings were as follows: positive electrospray mode, capillary voltage 3500 V, drying gas (N_2_) 12 L/min at 350°C, nebulizer gas (N_2_) pressure 50 psi, scan mode m/z 70–500, fragmentor voltage 150 V. The sample was dissolved in acetonitrile/water (1:1, containing 0.1% formic acid) at a concentration of 10 μg/mL. The injection volume was 1 μL, the flow rate was 0.80 mL/min and the column temperature was 30°C. The total run time was 25 min. The following gradient elution program was used: 0–2 min 2% B, followed by an increase to 60% within 15 min, then up to 80% within 20 min, returning to 2% within 25 min.

#### 2.2.5 ∣ Nuclear magnetic resonance spectroscopy (NMR)

The ECPLA sample was prepared in deuterated dimethyl sulfoxide (DMSO-d_6_) and ^1^H (600 MHz) and ^13^C (150 MHz) spectra were recorded on a Bruker Avance III 600 MHz NMR spectrometer (Coventry, UK). Spectra were referenced to residual solvent and assignments were supported by 1D and 2D experiments.

## 3 ∣ RESULTS AND DISCUSSION

The isomeric relationship between (2′*S*,4′*S*)-lysergic acid 2,4-dimethylazetidide (LSZ), and ECPLA (Figure 1) prompted the investigation of analytical features that can be used to differentiate between these two lysergamides. At the same time, it is worth noting that in addition to ECPLA and LSZ, several other isomeric lysergamides have been described in the scientific literature (although information about their availability or use as recreational drugs is not available). Three examples reflecting the C_21_H_25_N_3_O formula (MW 335.45 g/mol) include lysergic acid piperidide (LA-Pip), 1-methyl-*N*-pyrrolidyllysergamide (MPD-75), and lysergic acid cyclopentylamide (Cepentyl), respectively. Although some chemical and pharmacological descriptions of these three compounds are available (Table S1, Supporting Information), it is unclear whether they are likely to be the focus of future research and/or whether they will appear as new recreational drugs.

### 3.1 ∣ Gas chromatography–mass spectrometry (GC–MS)

**FIGURE 2:**
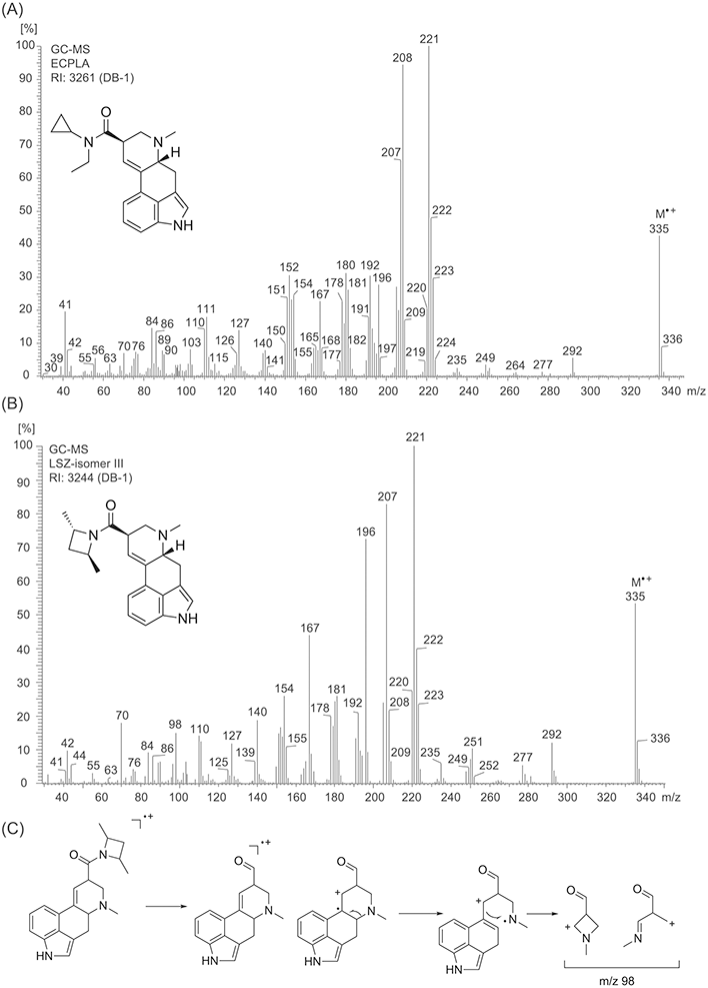
Electron ionization mass spectra. (A) ECPLA (B) LSZ. (C) Proposed formation of m/z 98

The electron ionization (EI) mass spectrum of ECPLA is depicted in Figure 2A. Proposed fragmentation pathways are shown as Supporting Information, based on previous investigations with a range of other lysergamides.^8-13^ Several fragment clusters that are commonly seen in the EI mass spectra of lysergamides (including ECPLA and LSZ) were observed, such as m/z 150–155, m/z 177–182, m/z 191–197, m/z 205–208, and m/z 219–224. The molecular ion was detected at m/z 335 with high relative abundance, which is typical for lysergamides. A comparison of match factor (MF) results between ECPLA and LSZ using the NIST database search function showed the following results: ECPLA (MF: 957, prob. 88.5%) and LSZ (MF = 835, prob. 4.38%). During the analysis of LSZ reported previously,^9^ two additional LSZ isomers were detected and their comparison with ECPLA resulted in values of MF = 822, prob. 2.83% (LSZ isomer I) and MF = 821, prob. 2.72% (Isomer II), respectively. The lysergamide LSM-775^11^ was suggested as a fifth hit (MF = 737, prob. 0.25%). In addition to the ECPLA/LSZ search results, a visual comparison of the EI mass spectra was considered helpful.

Apart from some shifts in relative abundance values, the EI mass spectrum of the isomeric LSZ^9^ (Figure 2B) showed comparable ions including the aforementioned fragment clusters and the formation of the retro-Diels-Alder fragment at m/z 292. One of the more noticeable differences in abundance in these lysergamide-related clusters included ions at m/z 196 (ECPLA: 28%; LSZ: 73%) and m/z 207/208 (ECPLA: 66/95%; LSZ: 83/22%). The base peaks were detected at m/z 221 in both cases. One minor but clear difference between the spectra of LSZ and ECPLA was the ion at m/z 98 (Figure 2B) which was not detected to that extent in related lysergamides investigated previously under the conditions used.^8-13^ Whilst the relative abundance of the ion was around 15% for LSZ, the abundance value was below 5% in the spectrum of ECPLA (Figure 2A). A proposed rationale for the formation of this ion is shown in Figure 2C. As shown in Figure 2, another minor difference was the ion at m/z 41, which was more abundant in the mass spectrum of ECPLA (19%) compared with LSZ (4%) (see the Supporting Information for a suggested mechanism). At the same time, it was found that gas chromatographic separation was also feasible, as indicated by the differences in the recorded retention parameters (ECPLA: RI = 3261: retention time = 28.82 min; LSZ - called isomer LSZ-III previously:^9^ RI = 3247: retention time = 27.41 min; LSZ-I: RI = 3174: retention time = 25.44 min; LSZ-II: RI = 3235: retention time = 27.06 min:^9^).

### 3.2 ∣ Liquid chromatography-mass spectrometry (LC–MS)

**FIGURE 3:**
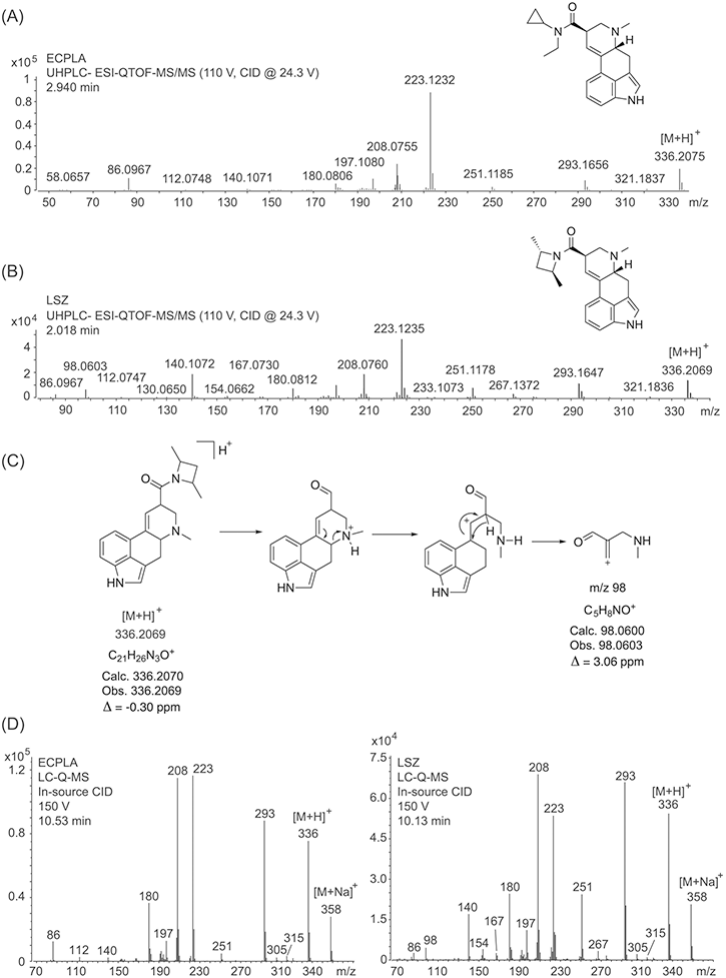
Electrospray ionization (ESI) quadrupole-time-of-flight tandem mass spectra. (A) ECPLA. (B) LSZ. (C) Proposed mechanism for the formation of the m/z 98 product ion. (D) Single quadrupole mass spectra of ECPLA (left) and LSZ (right) produced by in-source collision-induced dissociation

ESI-QTOF-MS/MS and ESI single quadrupole mass spectra subjected to in-source collision-induced dissociation are shown in Figure 3; proposed product ions involving high-resolution measurements are summarized in the Supporting Information based on the principles discussed in previous reports on related lysergamides.^8-13^ A visual comparison of mass spectral data (Figure 3) revealed that the product ions formed were comparable to the ions observed during LSZ analysis,^9^ although some variations were noted in the relative abundance of some of the product ions. For example, ions typically observed here and other related lysergamides^8-13^ included ions at m/z 251, 223, 208, 197, 180, and 140.

In the QTOF tandem mass spectrum of LSZ^9^ (Figure 3B), a product ion was observed at m/z 98 (C_5_H_8_NO^+^; calc. 98.0600; obs. 98.0603; Δ = 3.06 ppm), which did not appear in the tandem mass spectrum of ECPLA under the conditions used (Figure 3A). A suggested mechanism is shown in Figure 3C. A similar observation was made in the spectrum obtained from in-source CID under single quadrupole conditions, where m/z 98 was detected in the spectrum of LSZ but not ECPLA (Figure 3D). However, when a hybrid triple quadrupole linear ion trap mass spectrometer (QTRAP) was used (see the Supporting Information), the m/z 98 ion was not detected. Another minor feature common to all three types of ESI mass spectrometry was the detection of m/z 86, which appeared to be more pronounced in the three mass spectra of ECPLA (C_5_H_12_N^+^; calc. 86.0964; obs. 86.0967; Δ = 3.48 ppm) compared with LSZ, although the relatively low abundance might only offer limited support for distinguishing the two substances. Furthermore, an ion only observed in the mass spectrum of LSZ under these experimental conditions was noticeable at m/z 267 (C_16_H_17_N_3_O^•+^; calc. 267.1366; obs. 267.1372; Δ = 2.25 ppm, Figure 3B) that was particularly visible during LC-Q-MS analysis (Figure 3D). As shown in the Supporting Information, its detection might have reflected the formation of lysergic acid amide (LSA) as a radical cation.

An encouraging observation was that the three liquid chromatography-based techniques used in this study confirmed that ECPLA could be separated from LSZ. The retention times for ECPLA were 2.94 min (UHPLC-ESI-QTOF-MS/MS, Figure 3A), 10.53 min (HPLC-ESI-Q-MS, Figure 3D), and 6.72 min (HPLC-DAD, Supporting Information). In comparison, when LSZ was tested in a previous study under identical conditions, the relevant retention times were 2.018 min,^9^ 10.13 min,^9^ (Figure 3), and 6.14 min, respectively (Supporting Information). The UV full scan spectrum obtained from HPLC-DAD analysis (Supporting Information) confirmed that they were of limited value for differentiation purposes.

### 3.3 ∣ Spectroscopic features

**FIGURE 4:**
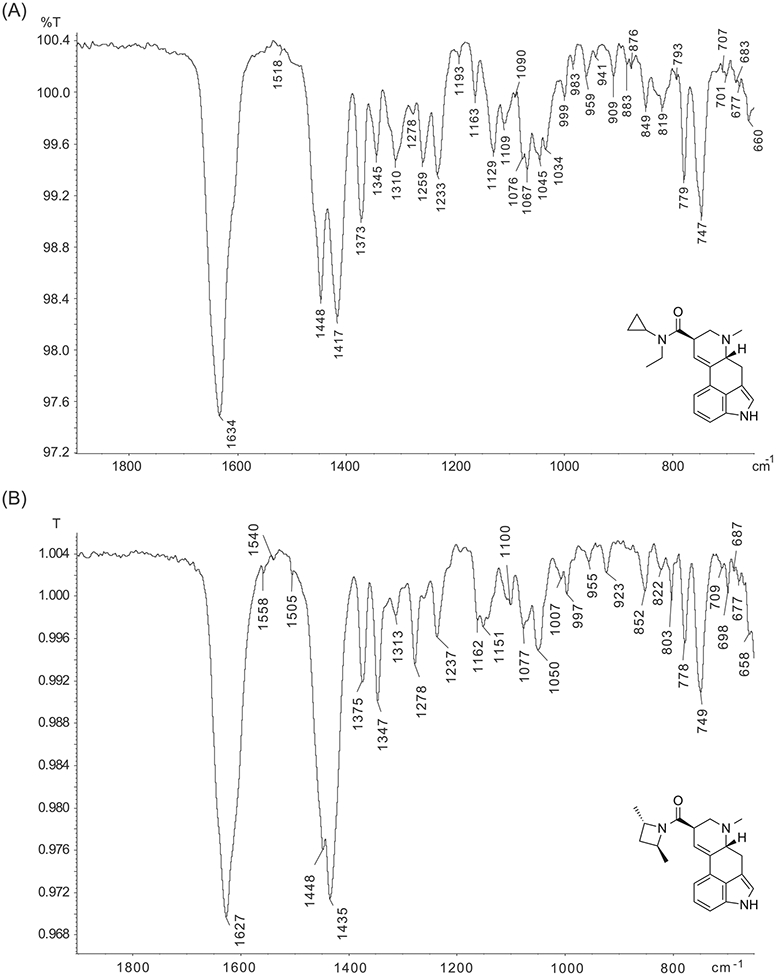
Partial spectra recorded by gas chromatography condensed phase infrared spectroscopy. (A) ECPLA. (B) LSZ bottom. Full spectra can be found as supporting information

A condensed-phase infrared (sIR) spectrum of ECPLA was obtained directly from the gas chromatographic peak (full spectrum shown as Supporting Information). Partial GC-sIR spectra recorded from ECPLA and LSZ^9^ are shown in Figure 4 for comparison. The carbonyl signal associated with the amide group was detected at 1634 cm^−1^ (ECPLA) or 1627 cm^−1^ (LSZ). However, some differences could also be observed, such as bands at 1448/1417 cm^−1^ (ECPLA) vs. 1448/1435 cm^−1^ (LSZ), 1233/1259 cm^−1^ (ECPLA) compared with 1237/1278 cm^−1^ (LSZ) and 1129 cm^−1^ (ECPLA) (Figure 4). Minor but distinct differences between various lysergamide homologs have been reported previously, including those detected in the region 1350–1150 cm^−1^.^14^ Indole N-H stretches at 3288 cm^−1^ (ECPLA) and 3263 cm^−1^ (LSZ), which are typically seen for lysergamides, were also detectable (see the Supporting Information). An advantage of using the GC-sIR system was that it also provided additional important data that facilitated the differentiation between ECPLA and LSZ on IR grounds in addition to relying on EI mass spectral features alone within a GC environment.

^1^H and ^13^C nuclear magnetic resonance (NMR) spectroscopy data obtained from 1D and 2D experiments (full spectra are available in the Supporting Information) and interpretations were consistent with previously published reports on other lysergamides^8-13^ (Table 1). The most pronounced difference related to the ring structures of ECPLA and LSZ^9^ was observed for the proton chemical shift of H-8α. In the case of ECPLA, this proton was detected as a multiplet between 4.34–4.33 ppm, whereas for LSZ, the multiplet was observed between 3.48–3.44 ppm:^9^ (see the Supporting Information for comparison). The downfield shift experienced by H-8α might have been caused by anisotropy in the cyclopropane moiety which led to some deshielding (C-8 chemical shift remained unaffected). The *N*-ethyl substituent in ECPLA was detected at 3.37 ppm (AB qq, *J*_20,20_ = 14.0, *J*_20,21_ = 7.0 Hz, 2H) (Table 1), whereas LSZ showed two methyl groups visible as two doublets at 1.35 ppm (*J* = 6.3 Hz, 3H) and 1.44 ppm (*J* = 6.2 Hz, 3H),^9^ respectively. Stacked spectra of ECPLA and LSZ have been included as Supporting Information for visual comparison. In the carbon NMR spectra, the most prominent differences between ECPLA and LSZ were observed in the carbon chemical shifts related to the amide substituents. For example, the carbon chemical shifts reflecting the 2,4-dimethylazetidide group in LSZ resonated at 55.74, 53.52, 22.51, 20.10, and 31.26 ppm,^9^ compared with 40.51, 13.18, 28.91, 8.98, and 8.59 ppm for the *N*-ethyl-*N*-cyclopropyl moiety in ECPLA (Table 1). The carbonyl carbon chemical shifts between the two isomers differed by ~2.6 ppm (ECPLA: 173.34 ppm; LSZ: 170.71 ppm^9^) (Supporting Information).

## 4 ∣ CONCLUSION

Recent preclinical investigations have shown that ECPLA produces LSD-like behavioral effects in mice, which suggests that it may act as a hallucinogenic drug in humans. Although ECPLA has not emerged as a new recreational drug, a proactive approach to the dissemination of key analytical data is warranted given the popularity of new LSD analogs on the marketplace for “designer drugs”. From a forensic and clinical perspective, there is also a need to differentiate between ECPLA and LSZ, which is a popular hallucinogen that emerged first in 2013. Mass spectral investigations of ECPLA and LSZ revealed some minor differences in the formation and abundance of specific ions. Implementation of gas chromatography-condensed phase-IR also revealed some distinctive differences between the two substances. ^1^H and ^13^C NMR spectroscopic investigations confirmed the differences in the two alkyl amide moieties and demonstrated that both substances could be separated using gas- and liquid chromatographic methods.

## Supplementary Material

Supplementary material

## Figures and Tables

**TABLE 1:** ^1^H and ^13^C NMR data for ECPLA hemitartrate in DMSO-d_6_ at 600/150 MHz

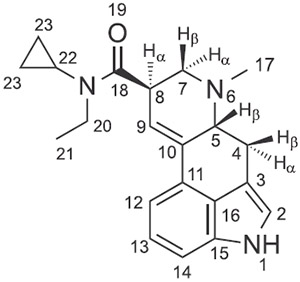		
No.	^13^C [δ/ppm]	^1^H [δ/ppm]
1	–	10.71 (s, 1H)
2	119.31	7.08–7.05 (m, 1H)^a^
3	108.61	–
4	26.52	3.51 (dd, *J* = 14.6, 5.5 Hz, 4β-H, 1H) 2.56–2.48 (m, 4α-H, 1H)^b^
5	62.67	3.16–3.14 (m, 5β-H, 1H)
6	–	–
7	55.19	3.10 (dd, *J* = 10.5, 4.1 Hz, 7α-H, 1H) 2.67 (*t*, *J* =10.3 Hz, 7β-H, 1H)
8	39.64	4.34–4.33 (m, 8α-H, 1H)
9	119.72	6.32 (s, 1H)
10	134.59	–
11	127.03	–
12	111.12	7.08–7.05 (m, 1H)^c^
13	122.27	7.08–7.05 (m, 1H)^d^
14	109.81	7.19 (d, *J* = 7.1 Hz, 1H)
15	133.83	–
16	125.74	–
17	43.20	2.53 (s, 3H)^e^
18	173.34	–
19	–	–
20	40.51	3.37 (AB qq, *J*_20,20_ = 14.0, *J*_20,21_ = 7.0 Hz, 2H)^f^
21	13.18	1.08 (t, *J* = 7.0 Hz, 3H)
22	28.91	2.94–2.90 (m, 1H)
23	8.98	0.95–0.91 (m, 2H)
23	8.59	0.87–0.82 (m, 2H)
TA ^g^	71.84	4.19 (s, 1H)
TA ^g^	173.54	–

a Overlapping with H-12 and H-13.

b Overlapping with 17-CH_3_ and solvent.

c Overlapping with H-2 and H-13.

d Overlapping with H-2 and H-12.

e Overlapping with 4α-H and solvent.

f Overlapping with residual H_2_O.

g TA, tartaric acid.
