# Supplementary material for: Analytical profile of N-ethyl-N-cyclopropyl lysergamide (ECPLA), an isomer of lysergic acid 2,4-dimethylazetidide (LSZ)
Source: Drug Test Anal. Author manuscript; Available in PMC 2022 Jun 13. (PMC9191644; doi:10.1002/dta.2911)
Supplement: Supplementary material [file NIHMS1802270-supplement-Supplementary_material.pdf]

Analytical profile of *N*-ethyl-*N*-cyclopropyl lysergamide (ECPLA), an isomer of lysergic acid 2,4-dimethylazetidide (LSZ)

Simon D. Brandt,[1,*] Pierce V. Kavanagh,[2] Folker Westphal,[3] Alexander Stratford,[4] Simon P. Elliott,[5] Geraldine Dowling,[2,6] Adam L. Halberstadt [7,8]

[1] *School of Pharmacy and Biomolecular Sciences, Liverpool John Moores University, Byrom Street, Liverpool L3 3AF, UK*

[2] *Department of Pharmacology and Therapeutics, School of Medicine, Trinity Centre for Health Sciences, St. James Hospital, Dublin 8, Ireland*

[3] *State Bureau of Criminal Investigation Schleswig-Holstein, Section Narcotics/Toxicology, Mühlenweg 166, D-24116 Kiel, Germany*

[4] *Synex Synthetics BV, Karveelweg 20, 6222NH, Maastricht, The Netherlands*

[5] *Elliott Forensic Consulting, Birmingham, UK*

[6] *Department of Life Sciences, School of Science, Sligo Institute of Technology, Ash Lane, Sligo, F91YW50, Ireland*

[7] *Department of Psychiatry, University of California San Diego, La Jolla, CA 92093-0804, USA*

[8] *Research Service, VA San Diego Healthcare System, La Jolla, CA, USA*

* Correspondence to: Simon D. Brandt, School of Pharmacy and Biomolecular Sciences, Liverpool John Moores University, Byrom Street, Liverpool, L3 3AF, UK. E-Mail: s.brandt@ljmu.ac.uk

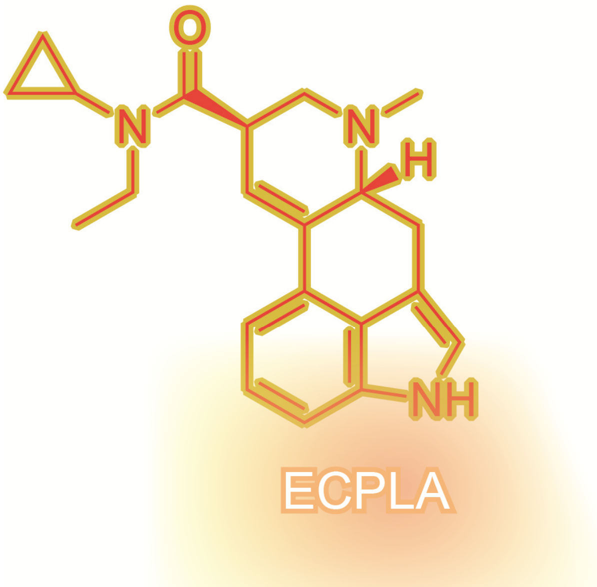

**Content**	**Page**
Proposed fragmentation pathways (electron ionization) for ECPLA	S3–4
Proposed fragmentation pathways (electrospray ionization) for ECPLA and LSZ (m/z 86 and m/z 267)	S5–S6
LC-MS/MS (QTRAP) and LC-DAD conditions	S7
LC-UV/DAD traces for ECPLA and LSZ	S7
LC-ESI-MS/MS data (QTRAP) for ECPLA and LSZ	S8
ATR-IR of ECPLA hemitartrate	S9
GC-sIR spectrum of ECPLA	S10
Comparison ECPLA hemitartrate (AT-IR) vs. ECPLA base (GC-sIR)	S11
Comparison ECPLA vs. LSZ (GC-sIR)	S12–S14
NMR data for ECPLA hemitartrate (d_6-DMSO)	S15–S31
NMR data for ECPLA hemitartrate (CD_3OD)	S32–S39
Comparison NMR data for ECPLA vs. LSZ (d_6-DMSO)	S40–S43
Table S1. References associated with isomers LA-Pip, MPD-75, and Cepentyl	S44–S46

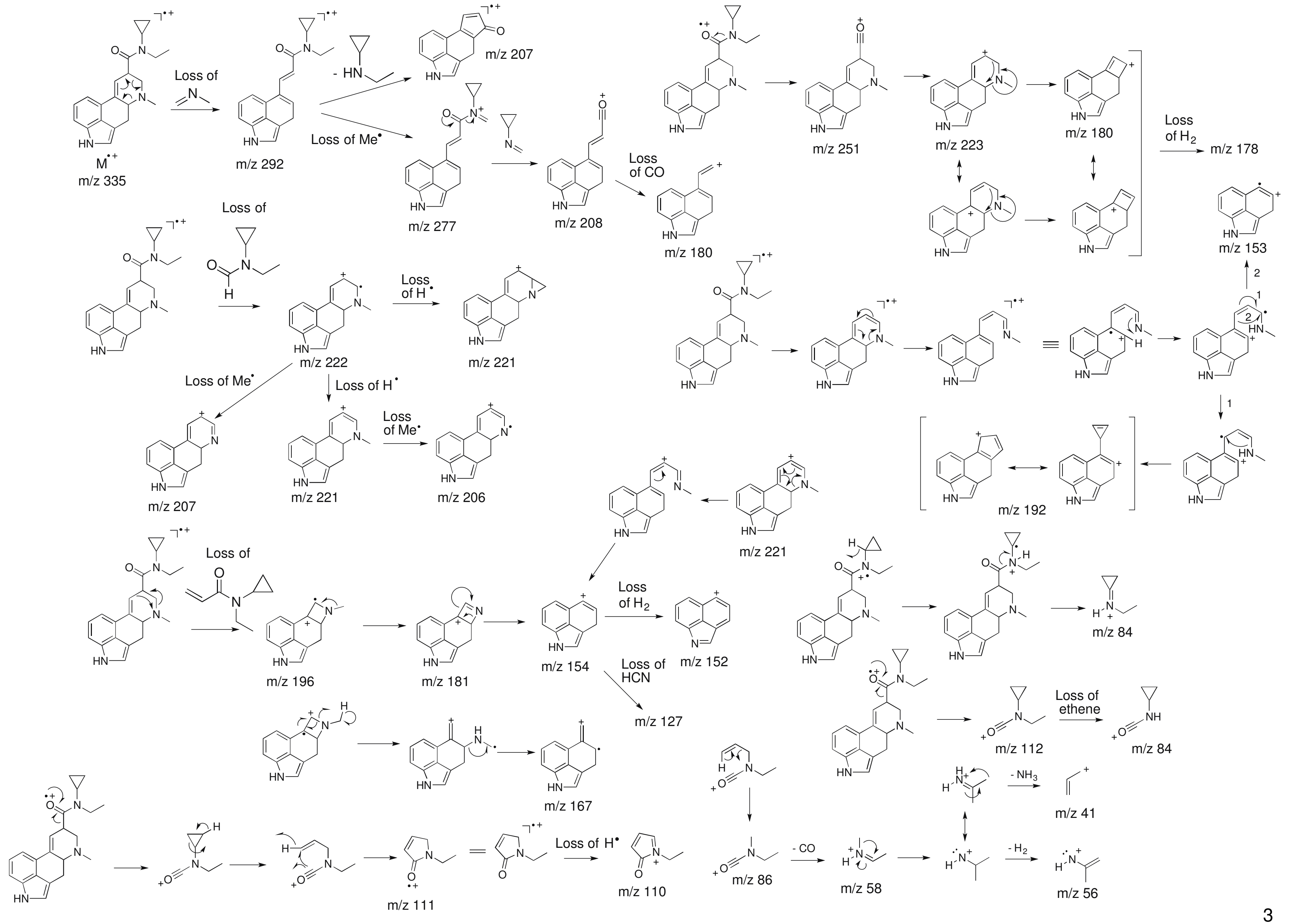
M•+
m/z 335
Loss of
m/z 292
- HN
m/z 207
Loss of Me•
m/z 277
m/z 208
Loss of CO
m/z 180
m/z 251
m/z 223
m/z 180
Loss of H2
m/z 178
m/z 153
Loss of
Loss of H•
m/z 222
m/z 221
Loss of Me•
Loss of H•
Loss of Me•
m/z 207
m/z 221
m/z 206
m/z 192
m/z 221
m/z 84
Loss of
m/z 196
m/z 181
m/z 154
Loss of H2
m/z 152
Loss of HCN
m/z 127
m/z 112
Loss of ethene
m/z 84
m/z 167
- NH3
m/z 41
m/z 111
Loss of H•
m/z 110
m/z 86
- CO
m/z 58
- H2
m/z 56

Alternative suggestions

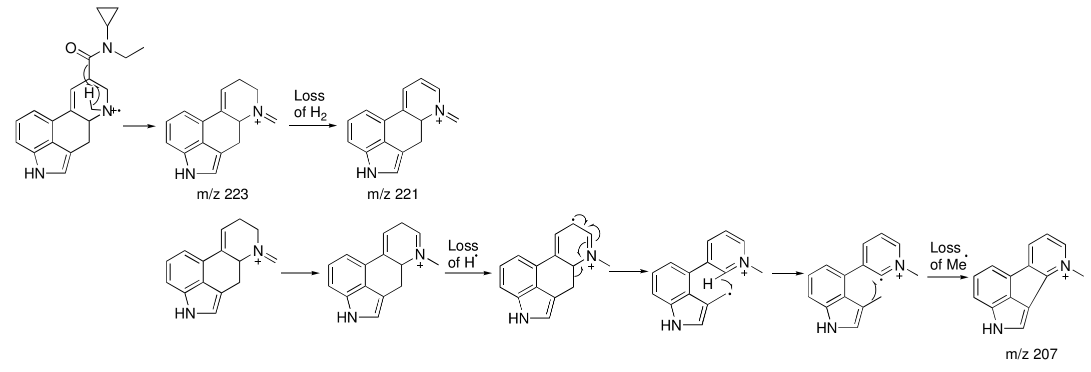

Adapted from: Nigam IC, Holmes JL. Mass spectrometry of lysergic acid diethylamide. *J Pharm Sci*. 1969;58(4):506-507

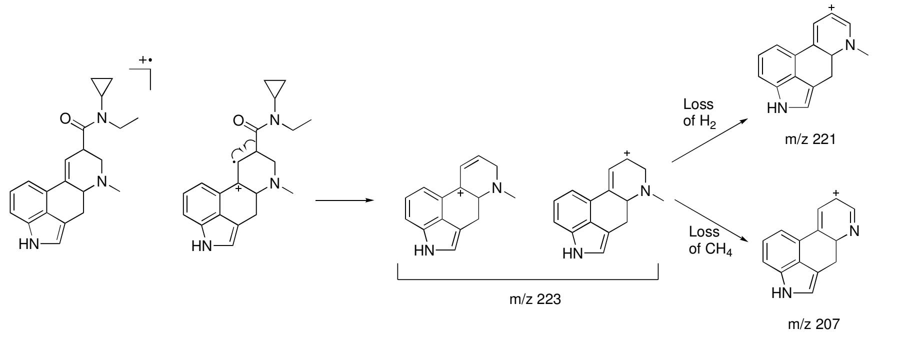

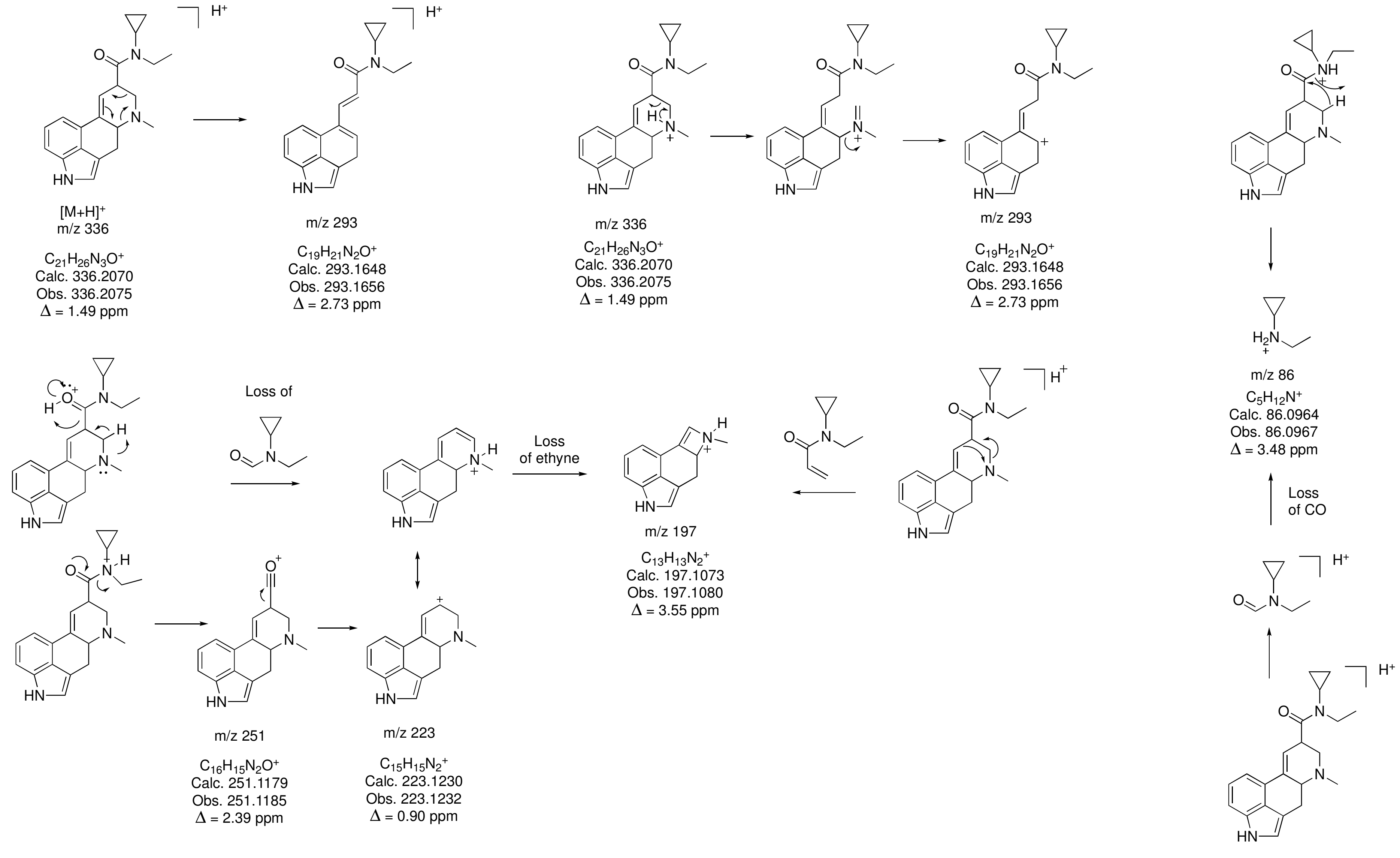
$[M+H]^+$
m/z 336
$C_{21}H_{26}N_3O^+$
Calc. 336.2070
Obs. 336.2075
Δ = 1.49 ppm
m/z 293
$C_{19}H_{21}N_2O^+$
Calc. 293.1648
Obs. 293.1656
Δ = 2.73 ppm
m/z 336
$C_{21}H_{26}N_3O^+$
Calc. 336.2070
Obs. 336.2075
Δ = 1.49 ppm
m/z 293
$C_{19}H_{21}N_2O^+$
Calc. 293.1648
Obs. 293.1656
Δ = 2.73 ppm
m/z 86
$C_5H_{12}N^+$
Calc. 86.0964
Obs. 86.0967
Δ = 3.48 ppm
Loss of CO
Loss of
Loss of ethyne
m/z 197
$C_{13}H_{13}N_2^+$
Calc. 197.1073
Obs. 197.1080
Δ = 3.55 ppm
m/z 251
$C_{16}H_{15}N_2O^+$
Calc. 251.1179
Obs. 251.1185
Δ = 2.39 ppm
m/z 223
$C_{15}H_{15}N_2^+$
Calc. 223.1230
Obs. 223.1232
Δ = 0.90 ppm

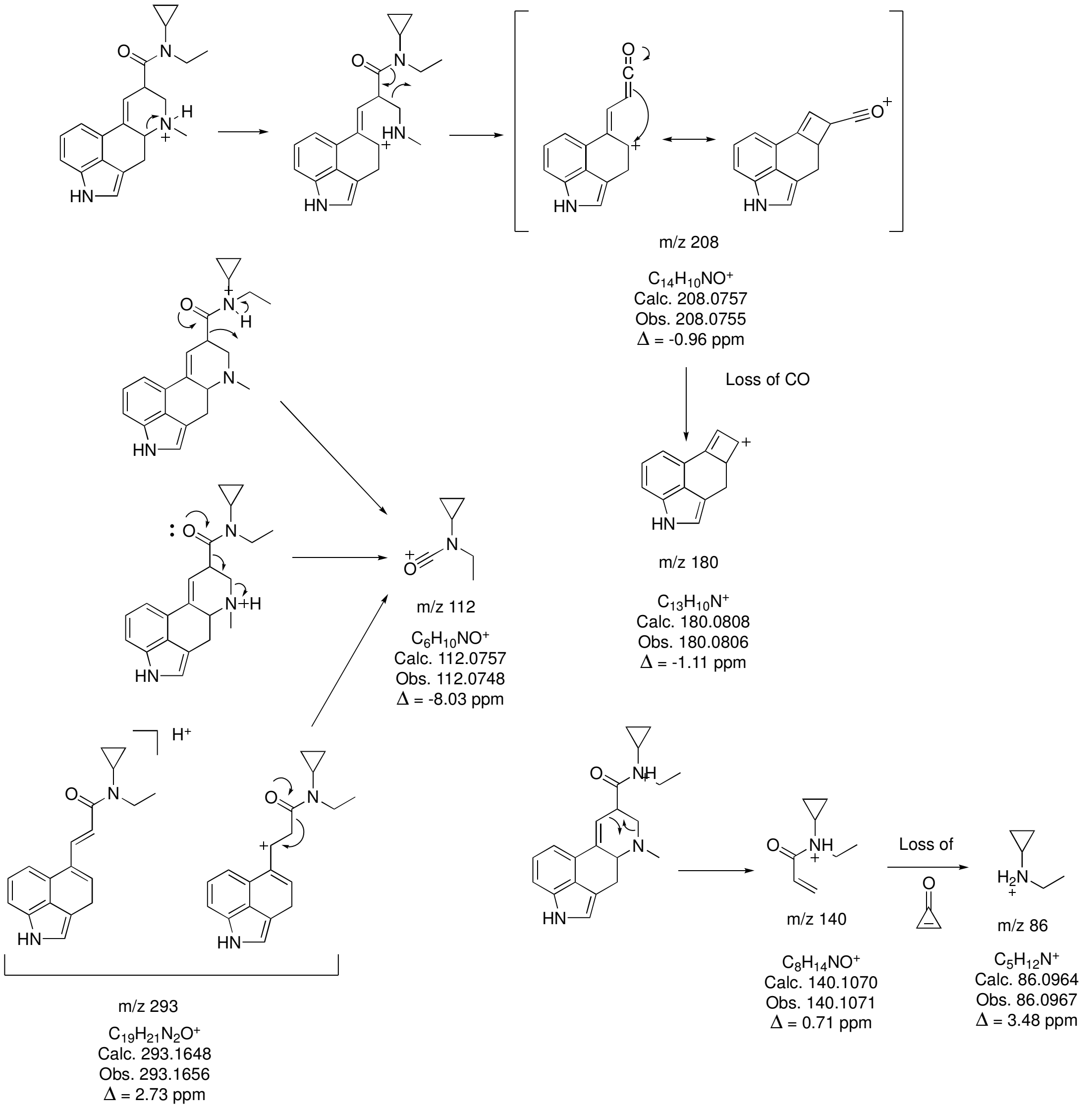
m/z 208
$C_{14}H_{10}NO^+$
Calc. 208.0757
Obs. 208.0755
Δ = -0.96 ppm
Loss of CO
m/z 180
$C_{13}H_{10}N^+$
Calc. 180.0808
Obs. 180.0806
Δ = -1.11 ppm
m/z 112
$C_6H_{10}NO^+$
Calc. 112.0757
Obs. 112.0748
Δ = -8.03 ppm
m/z 293
$C_{19}H_{21}N_2O^+$
Calc. 293.1648
Obs. 293.1656
Δ = 2.73 ppm
m/z 140
$C_8H_{14}NO^+$
Calc. 140.1070
Obs. 140.1071
Δ = 0.71 ppm
Loss of
m/z 86
$C_5H_{12}N^+$
Calc. 86.0964
Obs. 86.0967
Δ = 3.48 ppm

LSZ

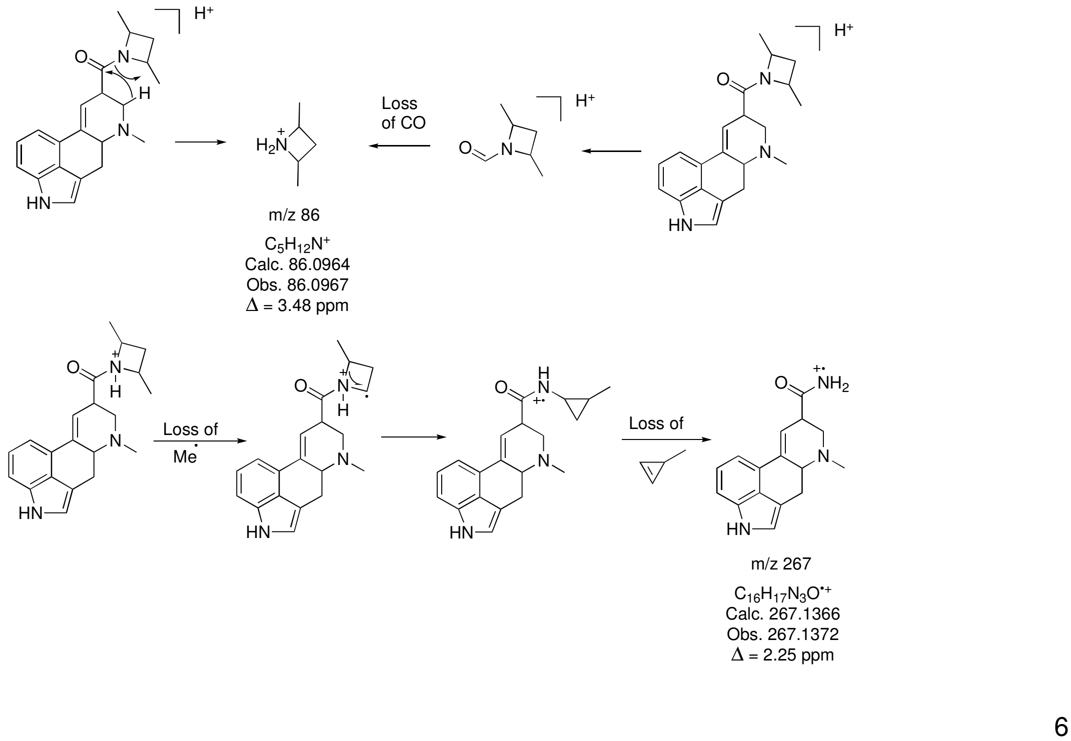
Loss of CO
m/z 86
$C_5H_{12}N^+$
Calc. 86.0964
Obs. 86.0967
Δ = 3.48 ppm
Loss of Me
Loss of
m/z 267
$C_{16}H_{17}N_3O^{\bullet+}$
Calc. 267.1366
Obs. 267.1372
Δ = 2.25 ppm

LC-MS/MS (QTRAP) and LC-DAD conditions

A Sciex 3200 QTRAP LC-MS/MS instrument was used coupled to an Agilent 1200 HPLC-DAD system (ABSciex, Cheshire, UK). A Phenomenex Gemini column (150 × 2.0 mm, 5 µm) was protected by a 4 mm × 3 mm Phenomenex Gemini guard column (Phenomenex, Cheshire, UK) using a mobile phase of 1 mM ammonium formate with 1% formic acid and acetonitrile (column temperature 30℃). Ionization was achieved with a Turbo V electrospray source. Liquid chromatography-mass spectrometry (LC-MS) data were obtained in positive enhanced mass spectrum (EMS) mode (scan range m/z 70–800) with information-dependent (above 10000 cps) enhanced product ion (EPI) scanning (between m/z 50–800). Product ions were formed using collision energies (CE) of 20, 30, and 50 eV in addition to collision energy spread (CES) of 35 V ± 15 eV. The following parameters were used: source temp: 500℃, curtain gas: 40, gas 1: 40 units, gas 2: 55 units, ion spray voltage: 5000 V, collision gas: high, declustering potential: 40 V, entrance potential: 5 V, scan rate: 1000 amu/s (EMS), 4000 amu/s (EPI) and LIT fill-time: 20 ms. LC-MS used a 3–19% acetonitrile gradient ramp in 5 min then up to 25% acetonitrile in 5 min followed by an increase up to 65% acetonitrile in 9 min and held for 1 min with a flow rate of 0.8 ml/min producing a run time 21 min, including 1 min equilibration returning to 3% acetonitrile.

ECPLA: LC-UV trace (6.72 min)

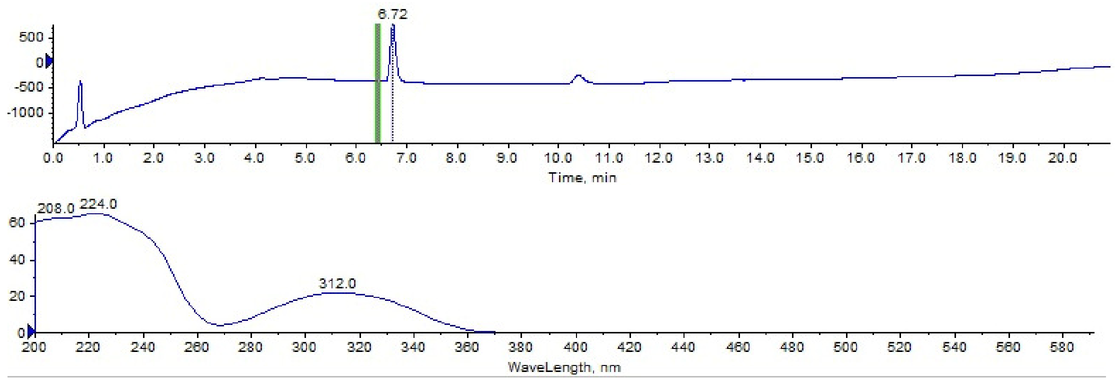

LSZ: LC-UV trace (6.14 min)

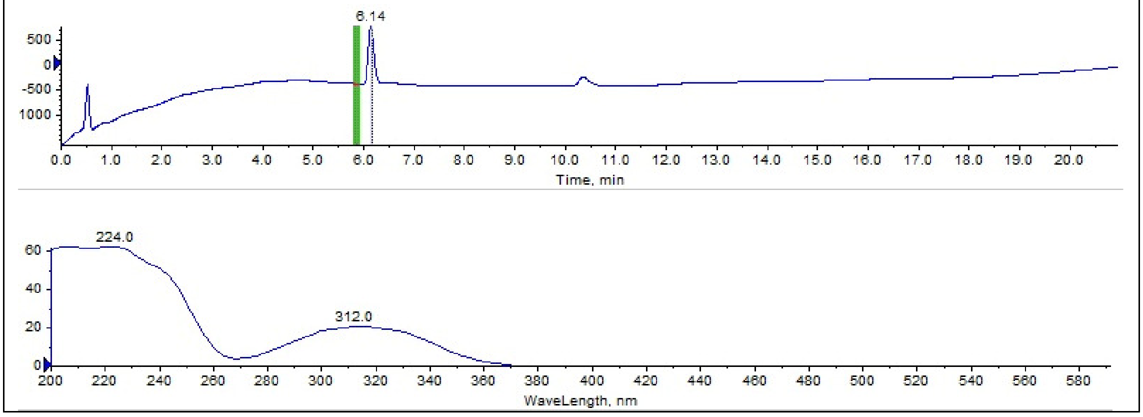

ECPLA: LC-MS/MS (QTRAP)

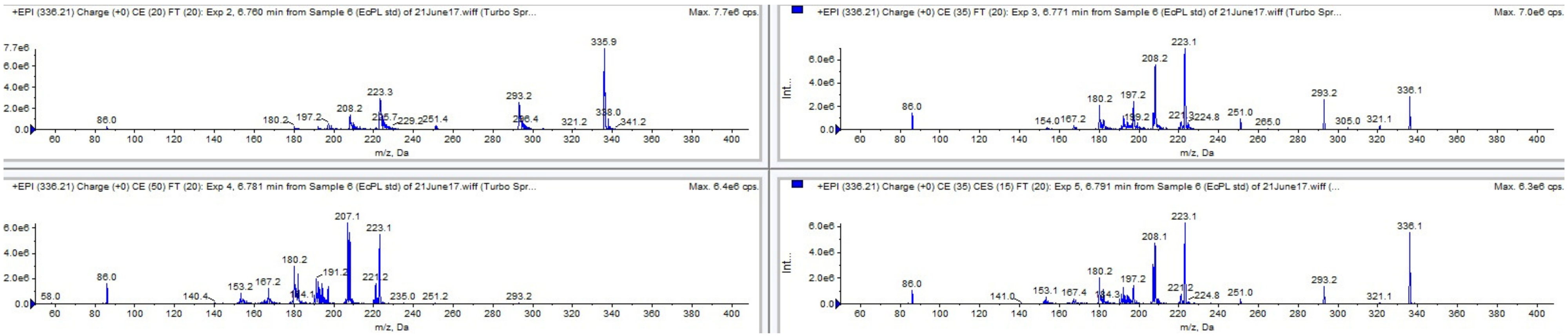

LSZ: LC-MS/MS (QTRAP)

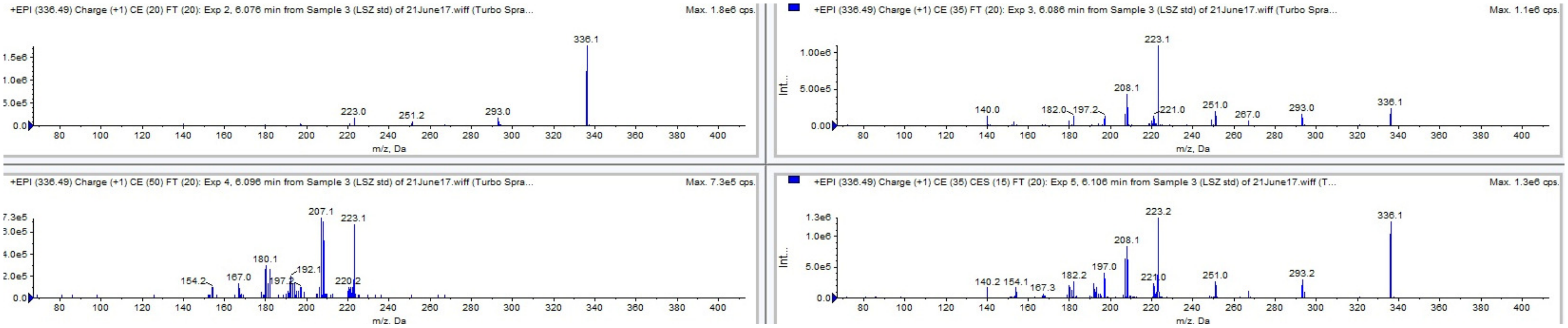

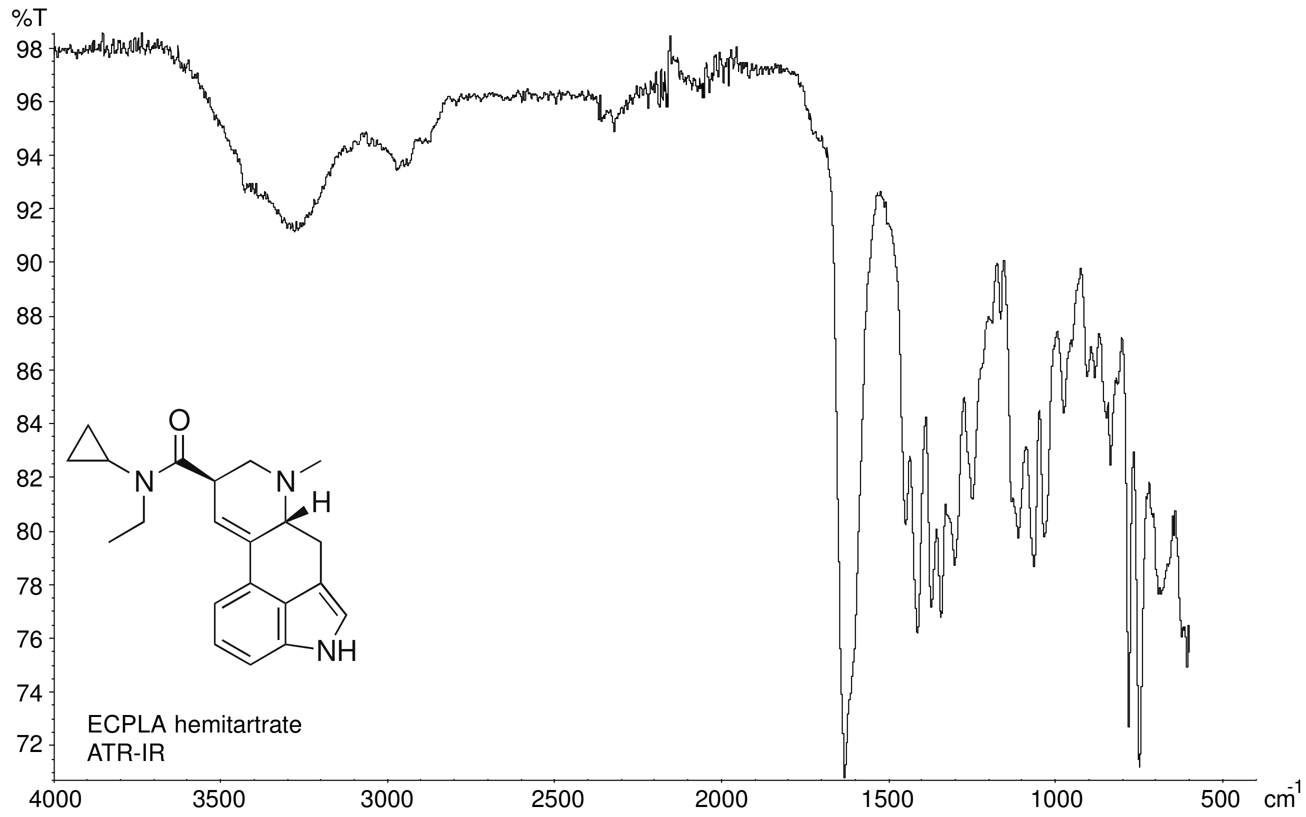
%T
98
96
94
92
90
88
86
84
82
80
78
76
74
72
O
N
N
H
NH
ECPLA hemitartrate
ATR-IR
4000
3500
3000
2500
2000
1500
1000
500
cm-1

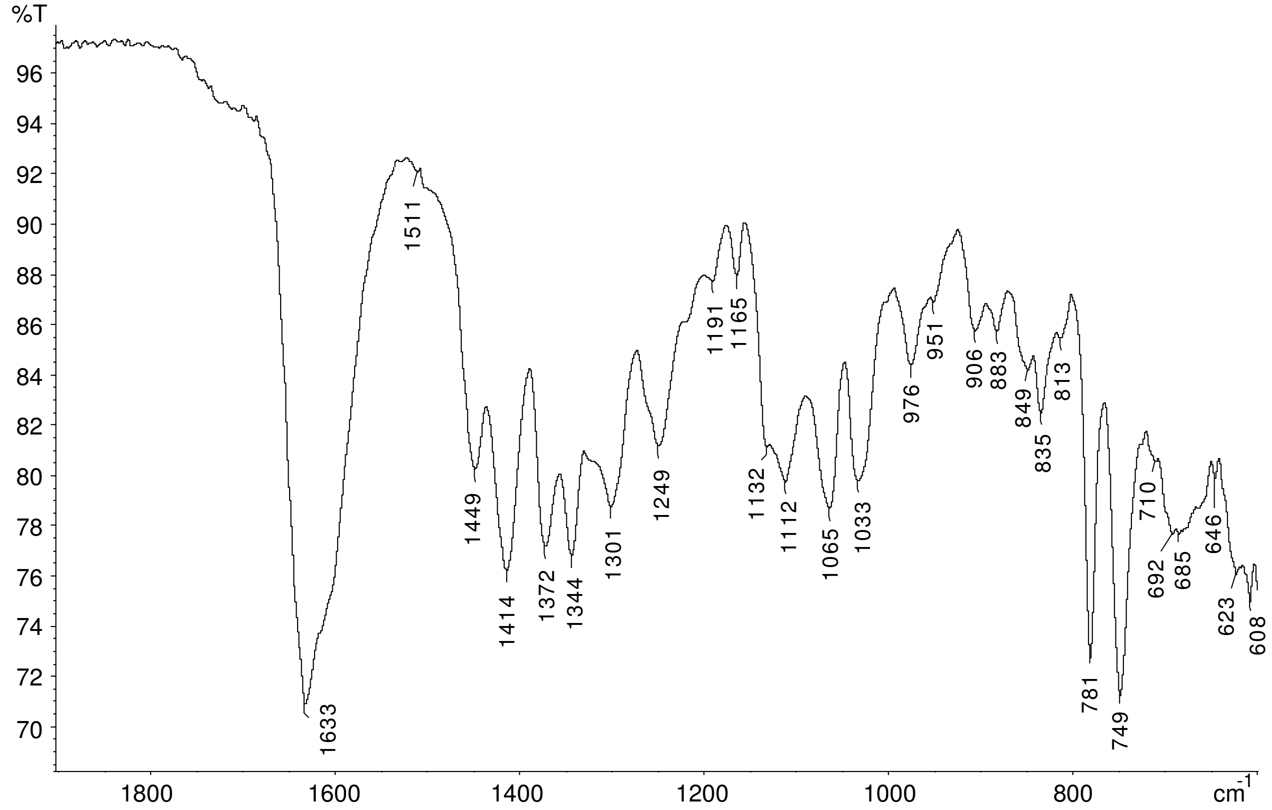
%T
96
94
92
90
88
86
84
82
80
78
76
74
72
70
1633
1511
1449
1414
1372
1344
1301
1249
1191
1165
1132
1112
1065
1033
976
951
906
883
849
835
813
781
749
710
692
685
646
623
608
1800
1600
1400
1200
1000
800
cm-1

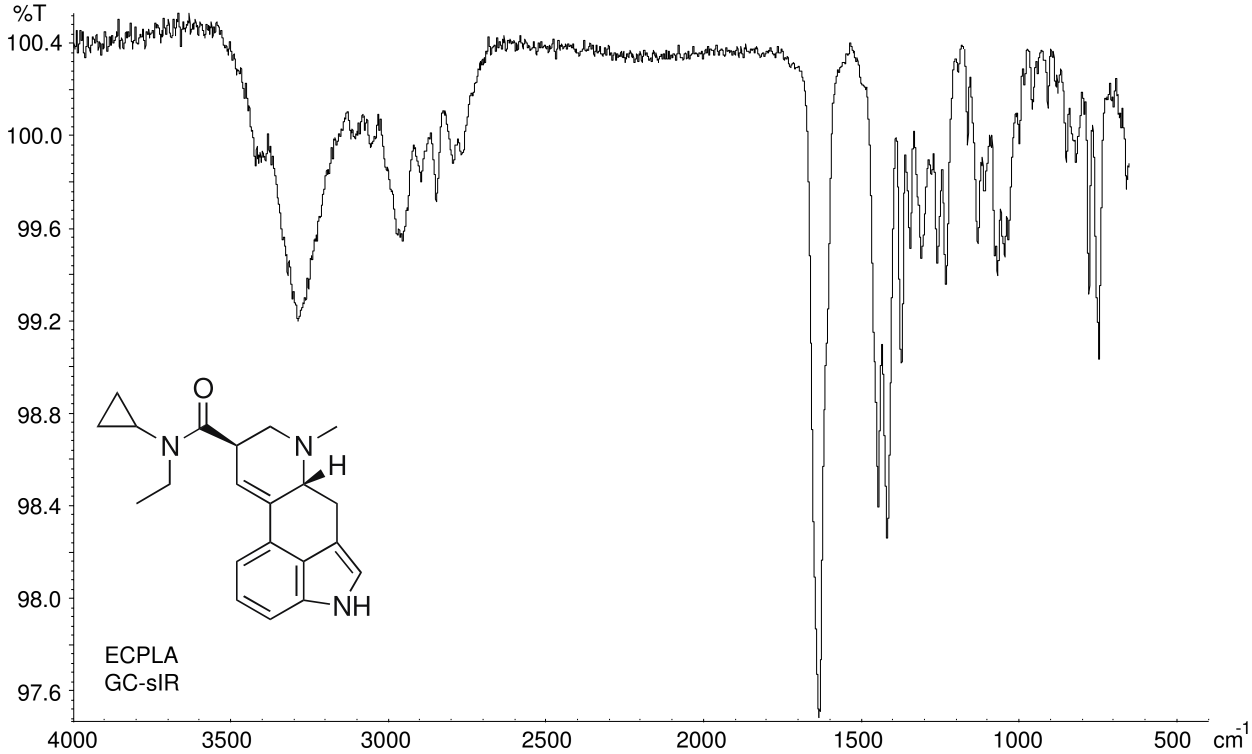
%T
100.4
100.0
99.6
99.2
98.8
98.4
98.0
97.6
4000
3500
3000
2500
2000
1500
1000
500
cm-1
ECPLA
GC-sIR
O
N
N
H
NH

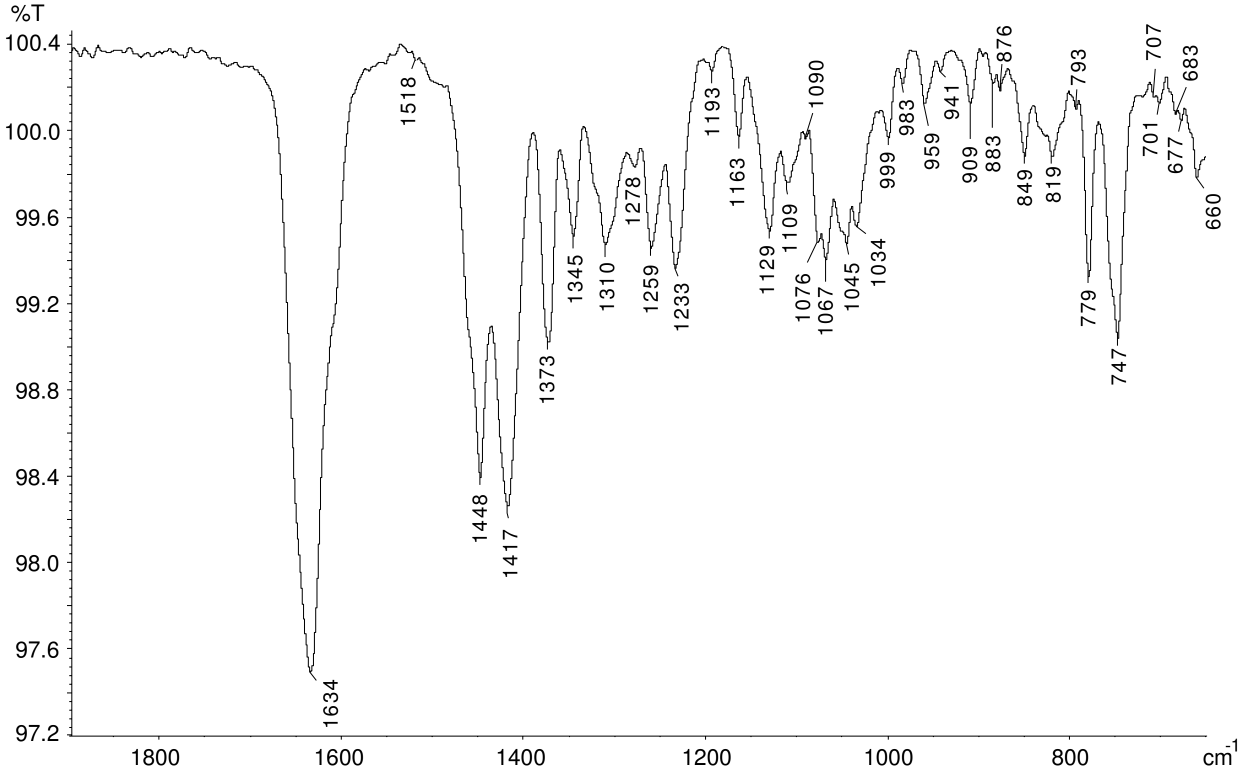
%T
100.4
100.0
99.6
99.2
98.8
98.4
98.0
97.6
97.2
1800
1600
1400
1200
1000
800
cm-1
1634
1518
1448
1417
1373
1345
1310
1278
1259
1233
1193
1163
1129
1109
1090
1076
1067
1045
1034
999
983
959
941
909
883
876
849
819
793
779
747
707
701
683
677
660

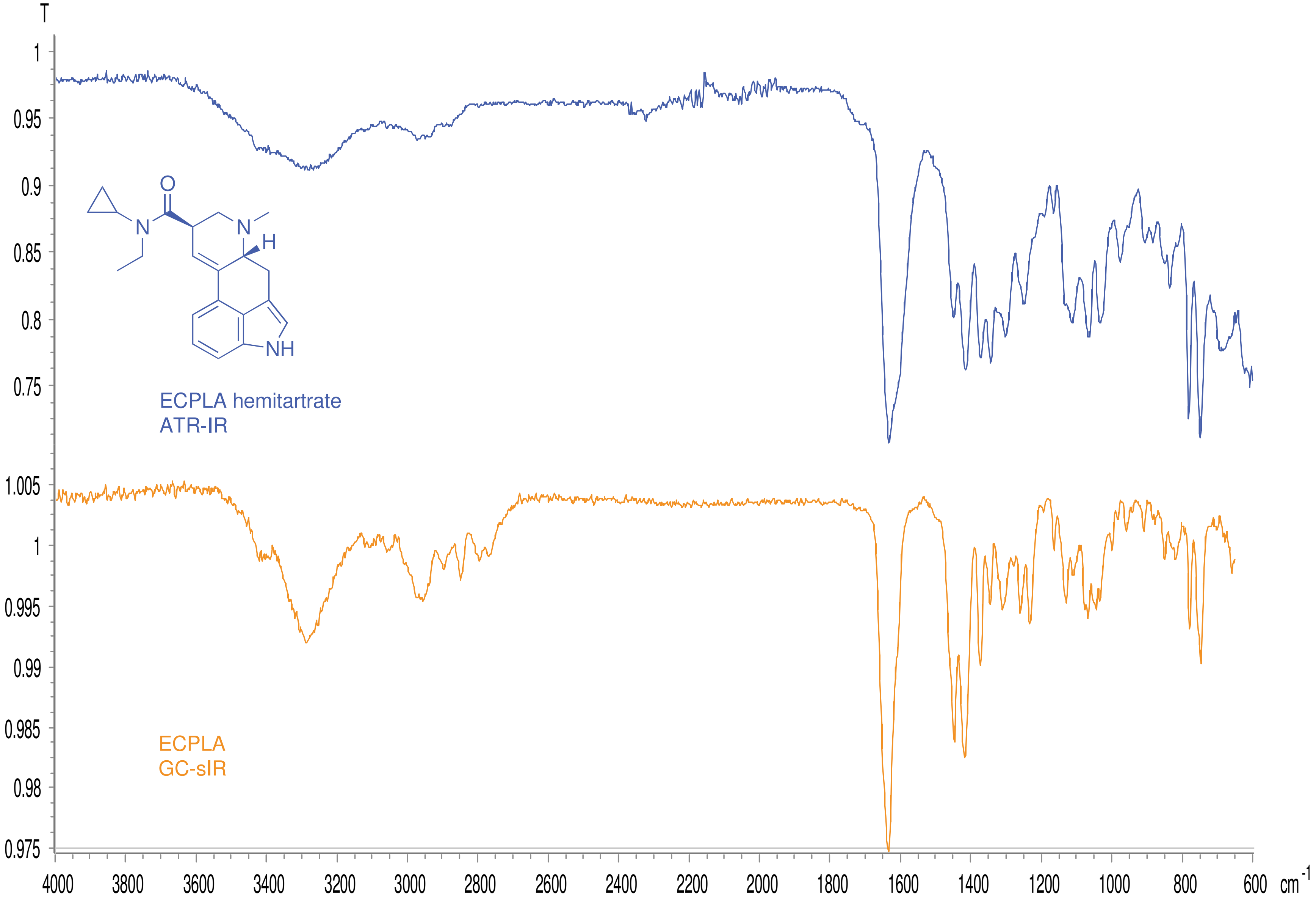
T
1
0.95
0.9
0.85
0.8
0.75
O
N
N
H
NH
ECPLA hemitartrate
ATR-IR
1.005
1
0.995
0.99
0.985
0.98
0.975
ECPLA
GC-sIR
4000
3800
3600
3400
3200
3000
2800
2600
2400
2200
2000
1800
1600
1400
1200
1000
800
600
cm-1

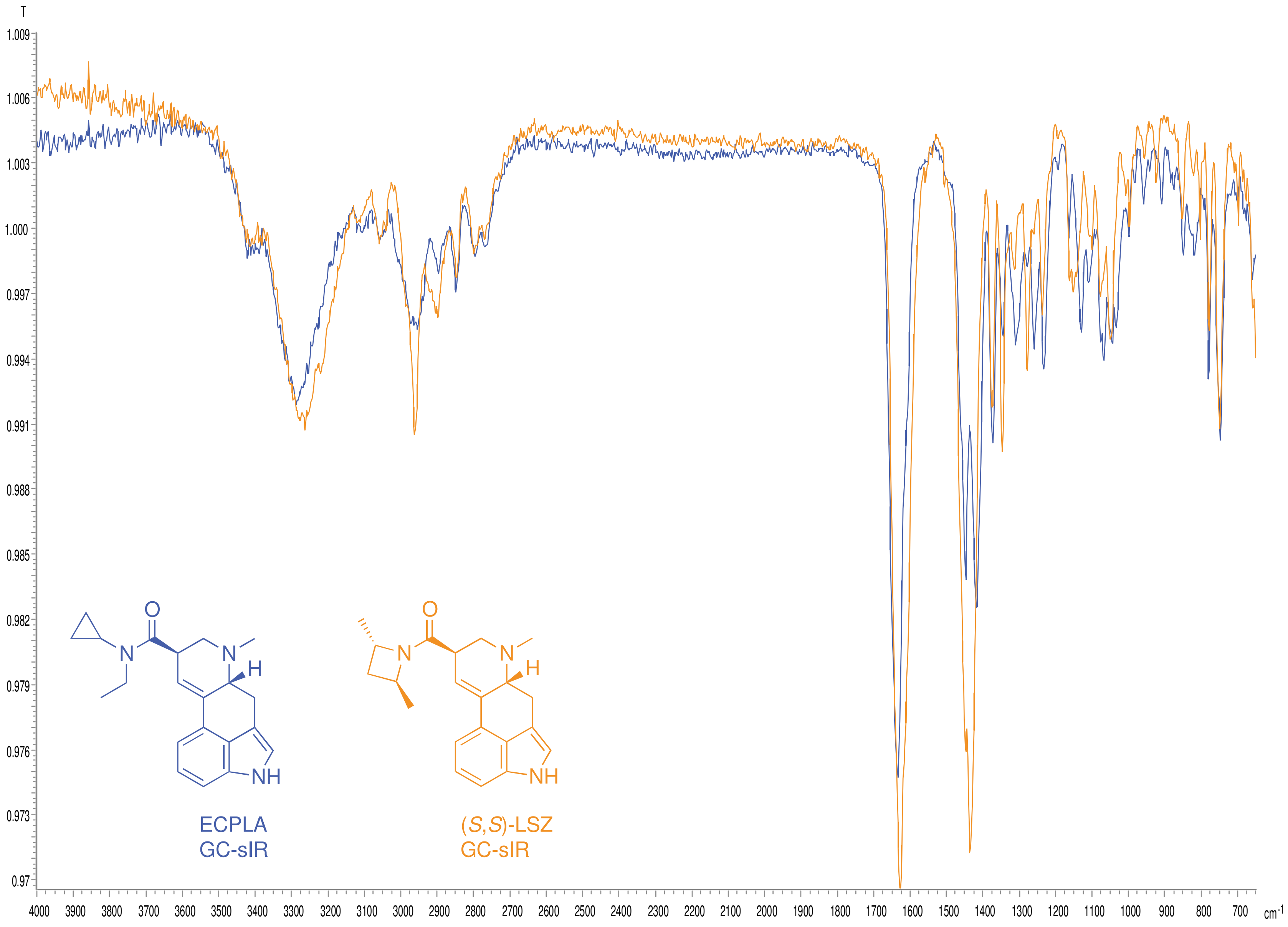
T
1.009
1.006
1.003
1.000
0.997
0.994
0.991
0.988
0.985
0.982
0.979
0.976
0.973
0.97
4000 3900 3800 3700 3600 3500 3400 3300 3200 3100 3000 2900 2800 2700 2600 2500 2400 2300 2200 2100 2000 1900 1800 1700 1600 1500 1400 1300 1200 1100 1000 900 800 700
cm-1
ECPLA
GC-sIR
(S,S)-LSZ
GC-sIR

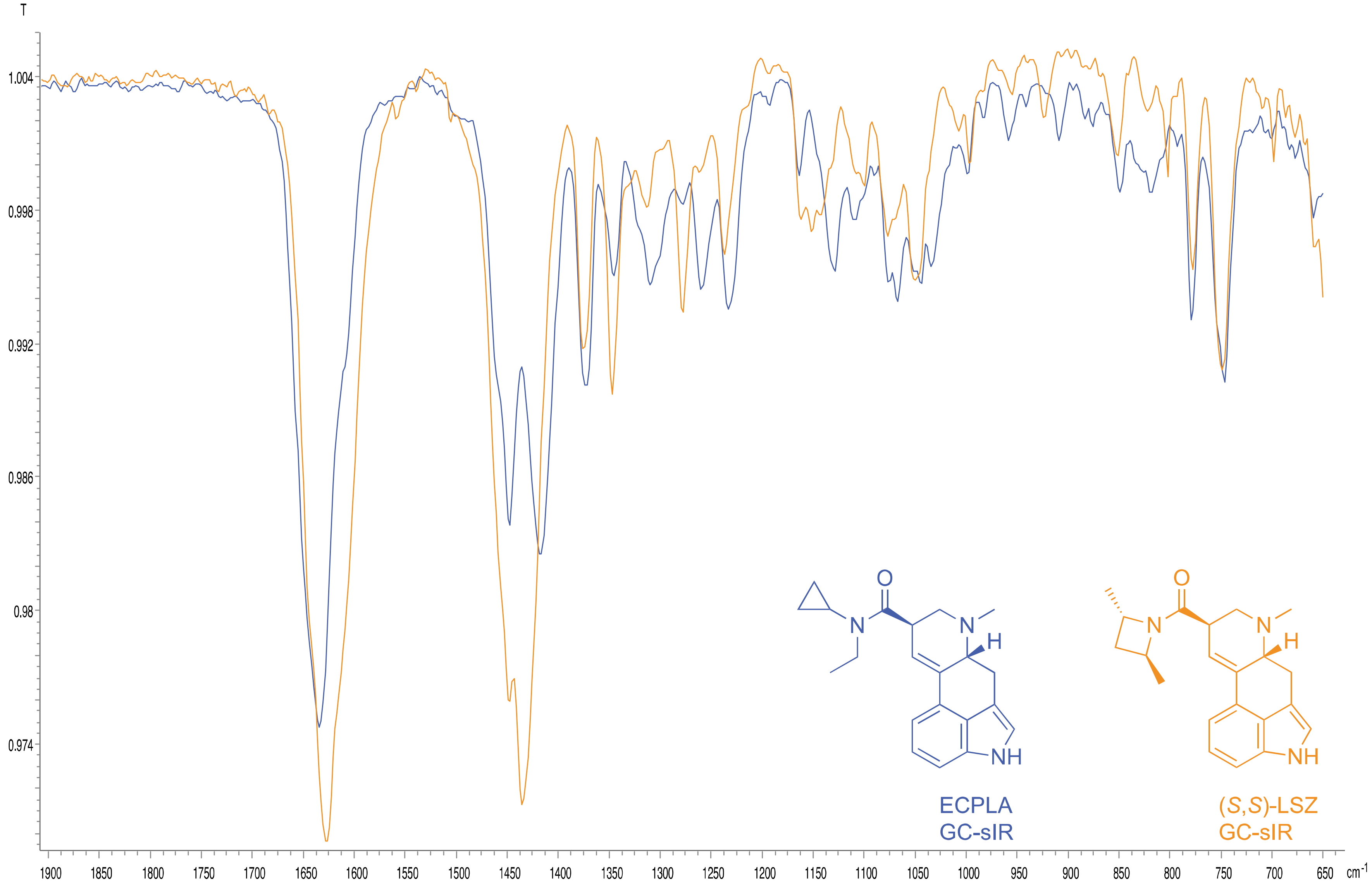
T
1.004
0.998
0.992
0.986
0.98
0.974
1900 1850 1800 1750 1700 1650 1600 1550 1500 1450 1400 1350 1300 1250 1200 1150 1100 1050 1000 950 900 850 800 750 700 650 cm-1
ECPLA
GC-sIR
(S,S)-LSZ
GC-sIR

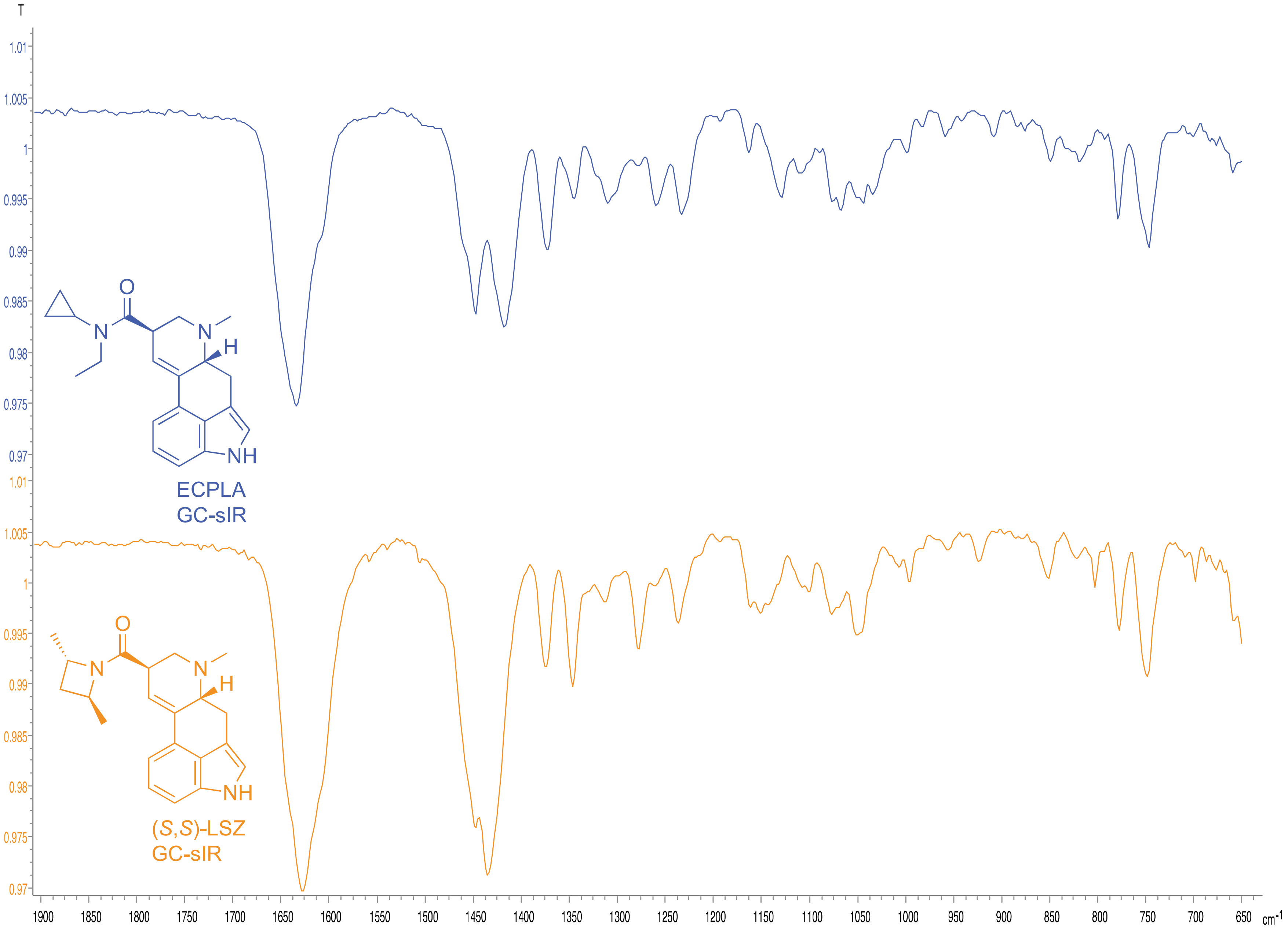
T
ECPLA
GC-sIR
(S,S)-LSZ
GC-sIR
cm-1

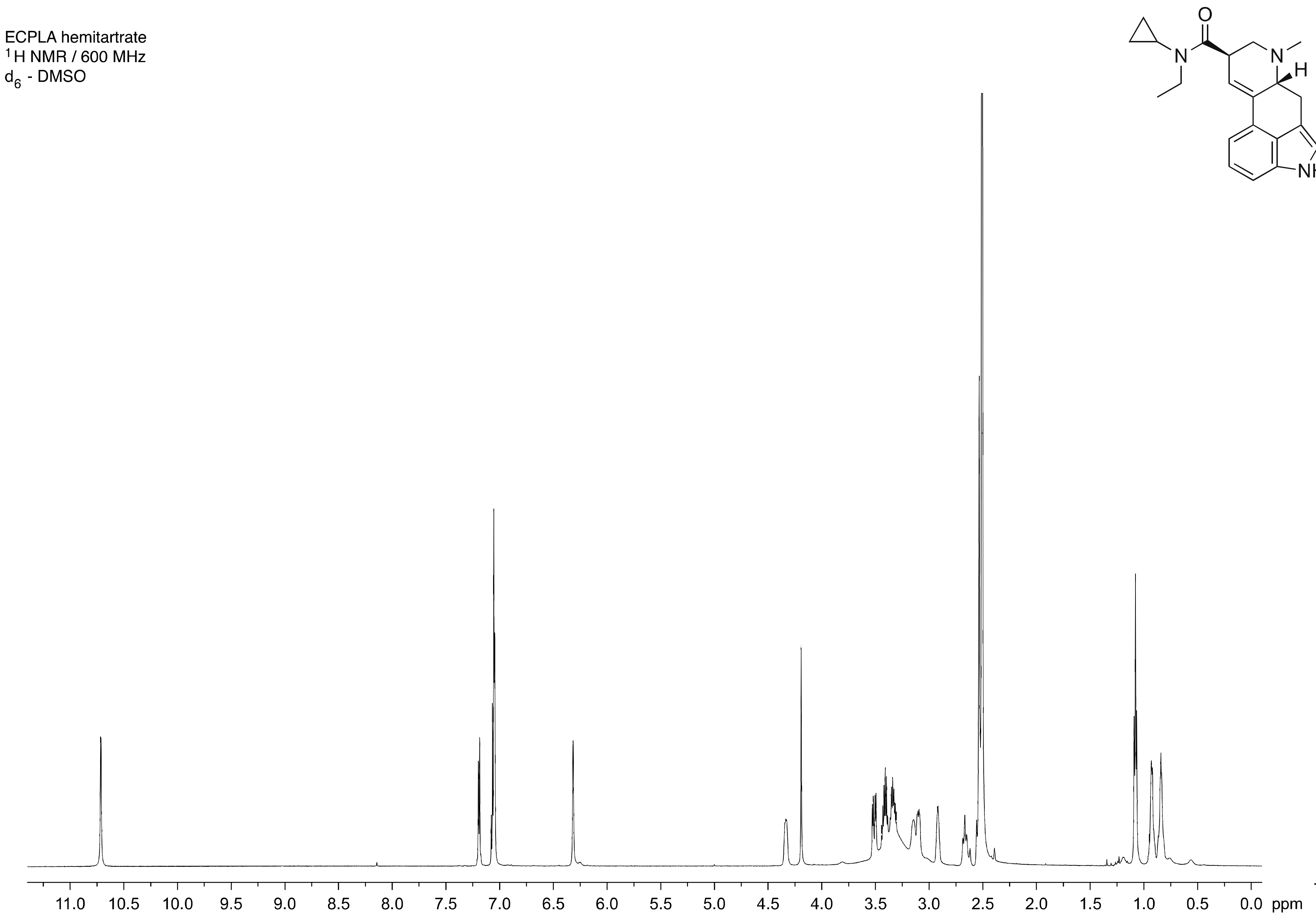
ECPLA hemitartrate
1H NMR / 600 MHz
d6 - DMSO
O
N
N
H
NH
11.0 10.5 10.0 9.5 9.0 8.5 8.0 7.5 7.0 6.5 6.0 5.5 5.0 4.5 4.0 3.5 3.0 2.5 2.0 1.5 1.0 0.5 0.0 ppm

ECPLA hemitartrate
^{1}H NMR / 600 MHz
d_6 - DMSO

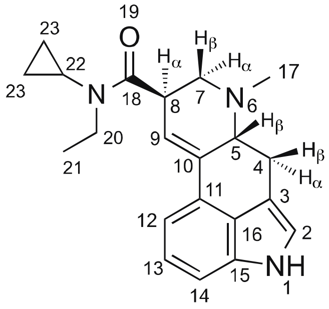

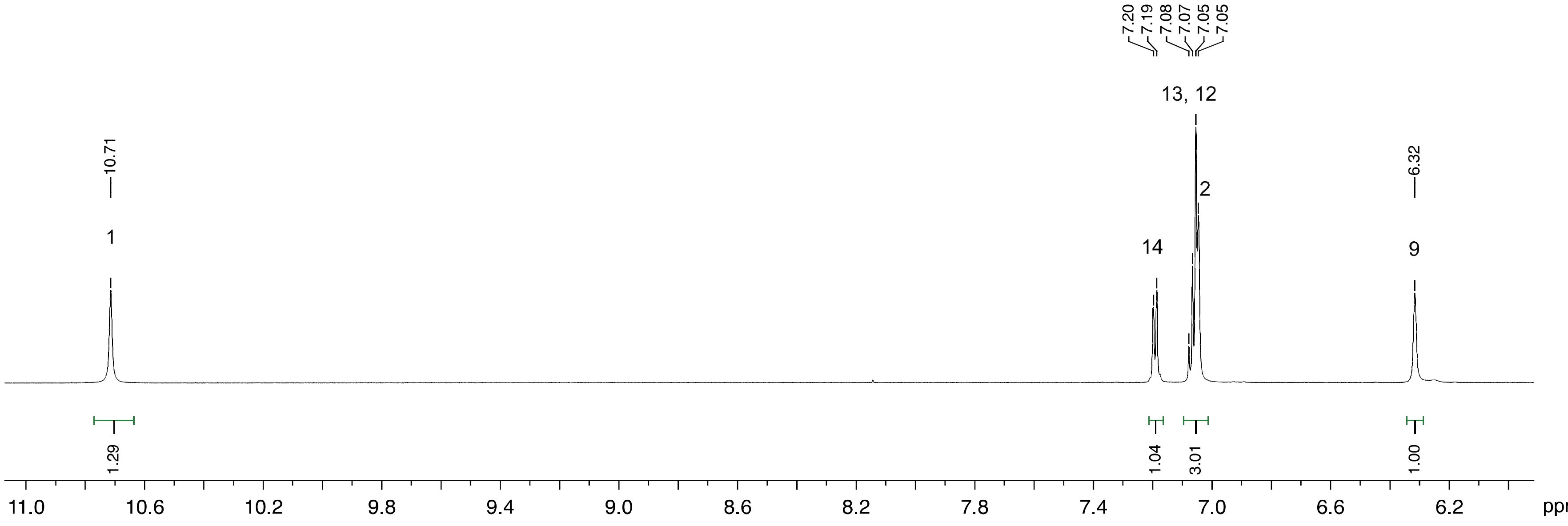

ECPLA hemitartrate
^{1}H NMR / 600 MHz
d_6 - DMSO

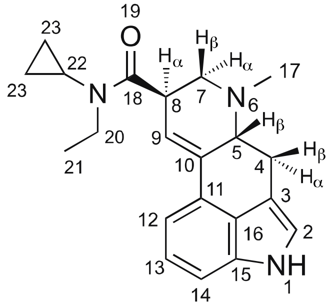

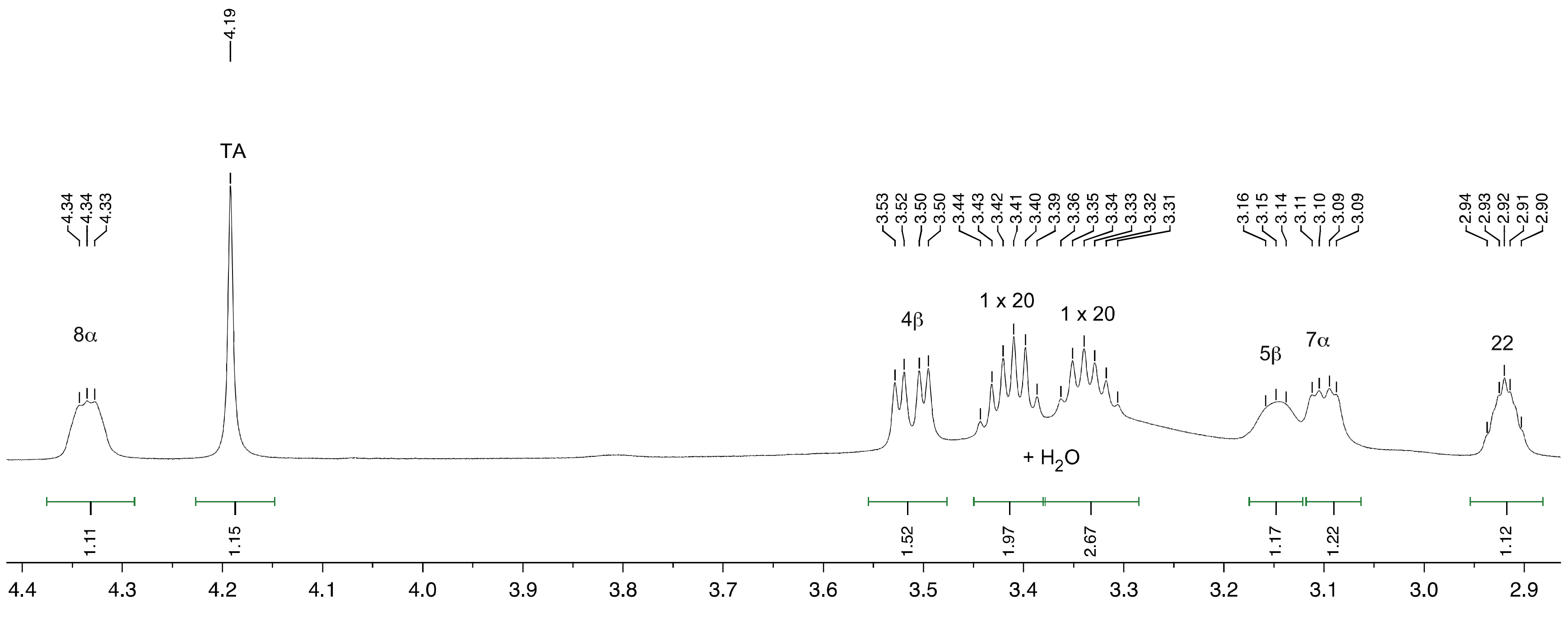

TA = tartaric acid

ECPLA hemitartrate
^{1}H NMR / 600 MHz
d_6 - DMSO

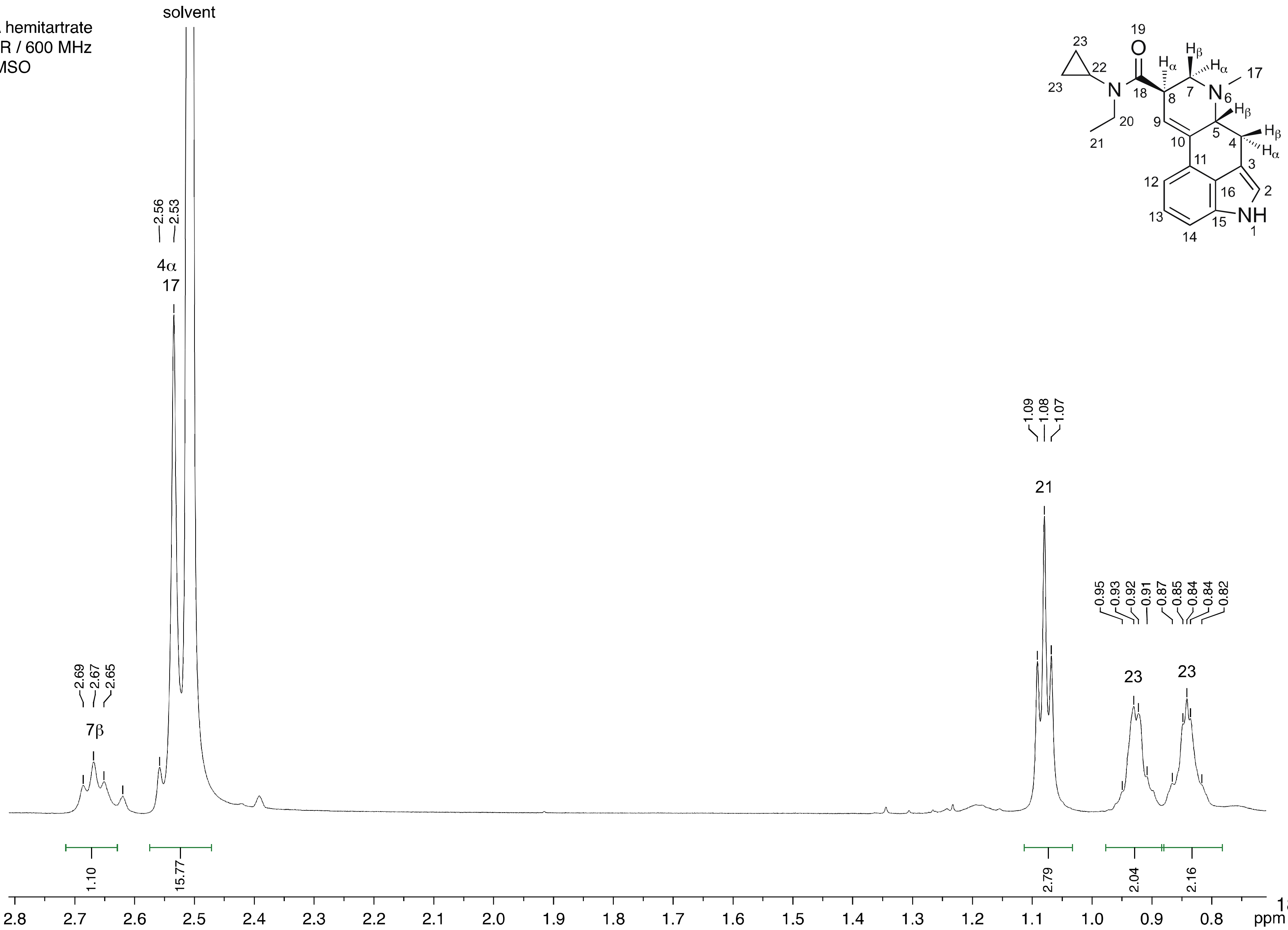

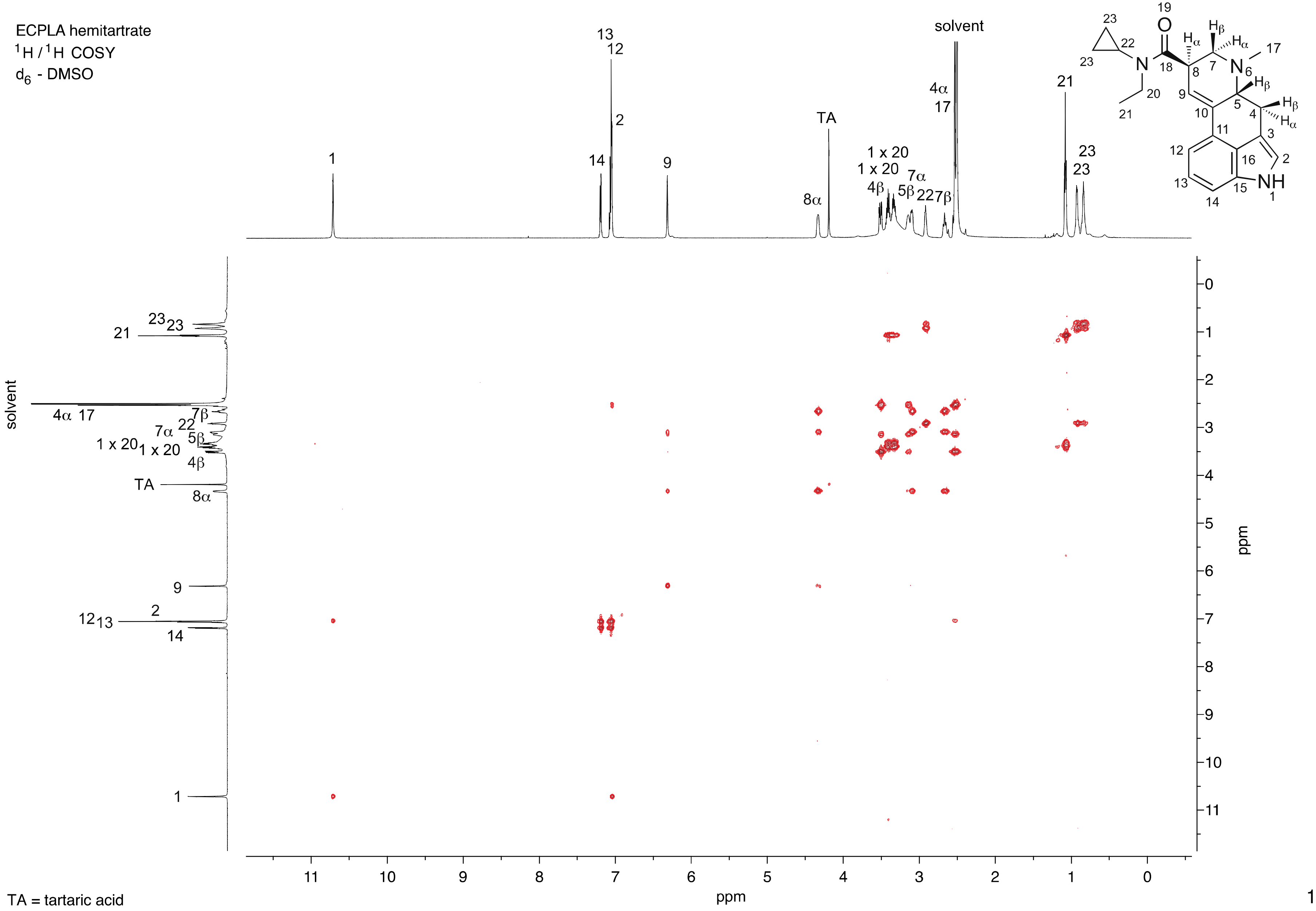

TA = tartaric acid

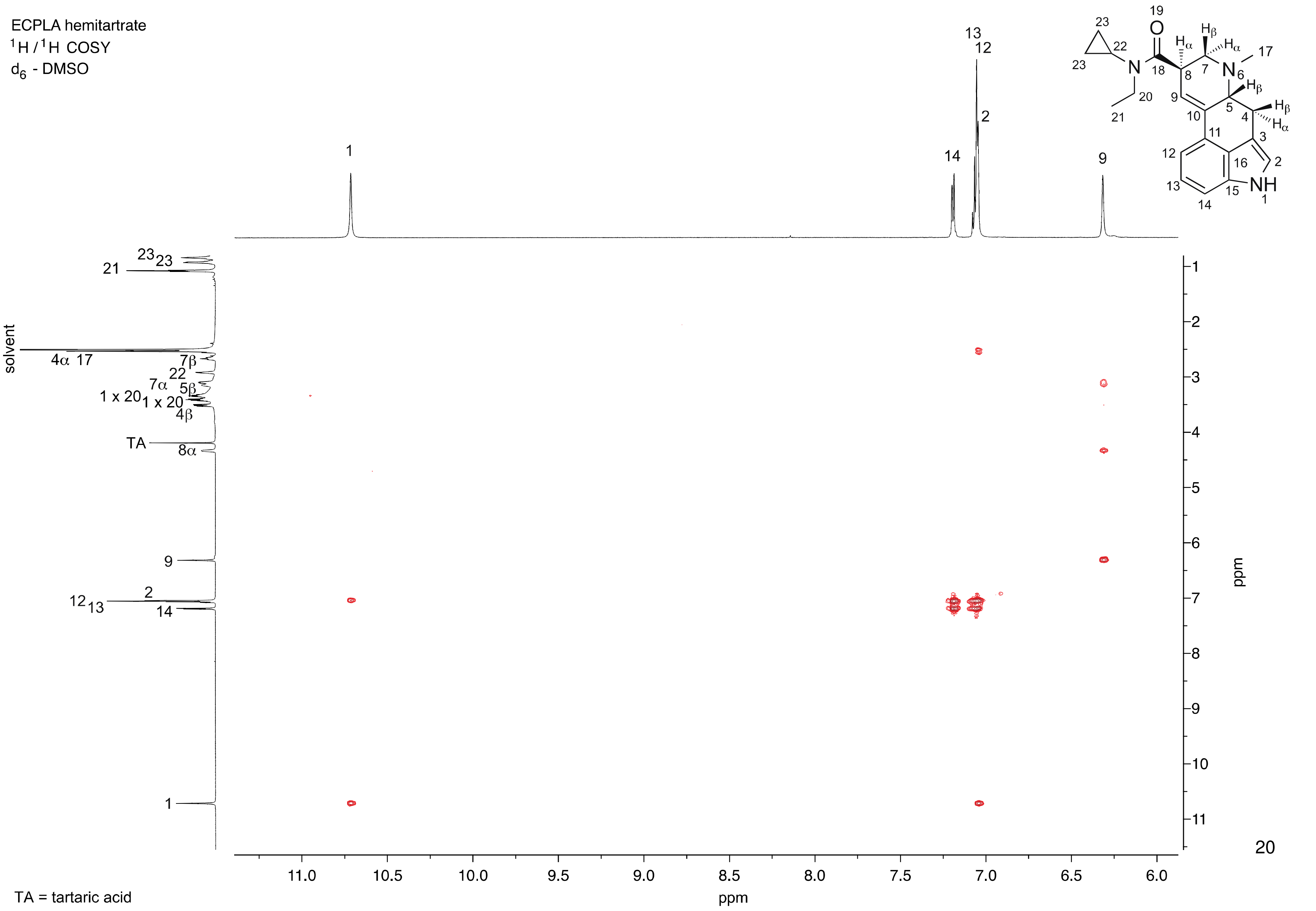
ECPLA hemitartrate
1H / 1H COSY
d6 - DMSO
solvent
TA
ppm
TA = tartaric acid

ECPLA hemitartrate
$^1H/^1H$ COSY
d_6 - DMSO

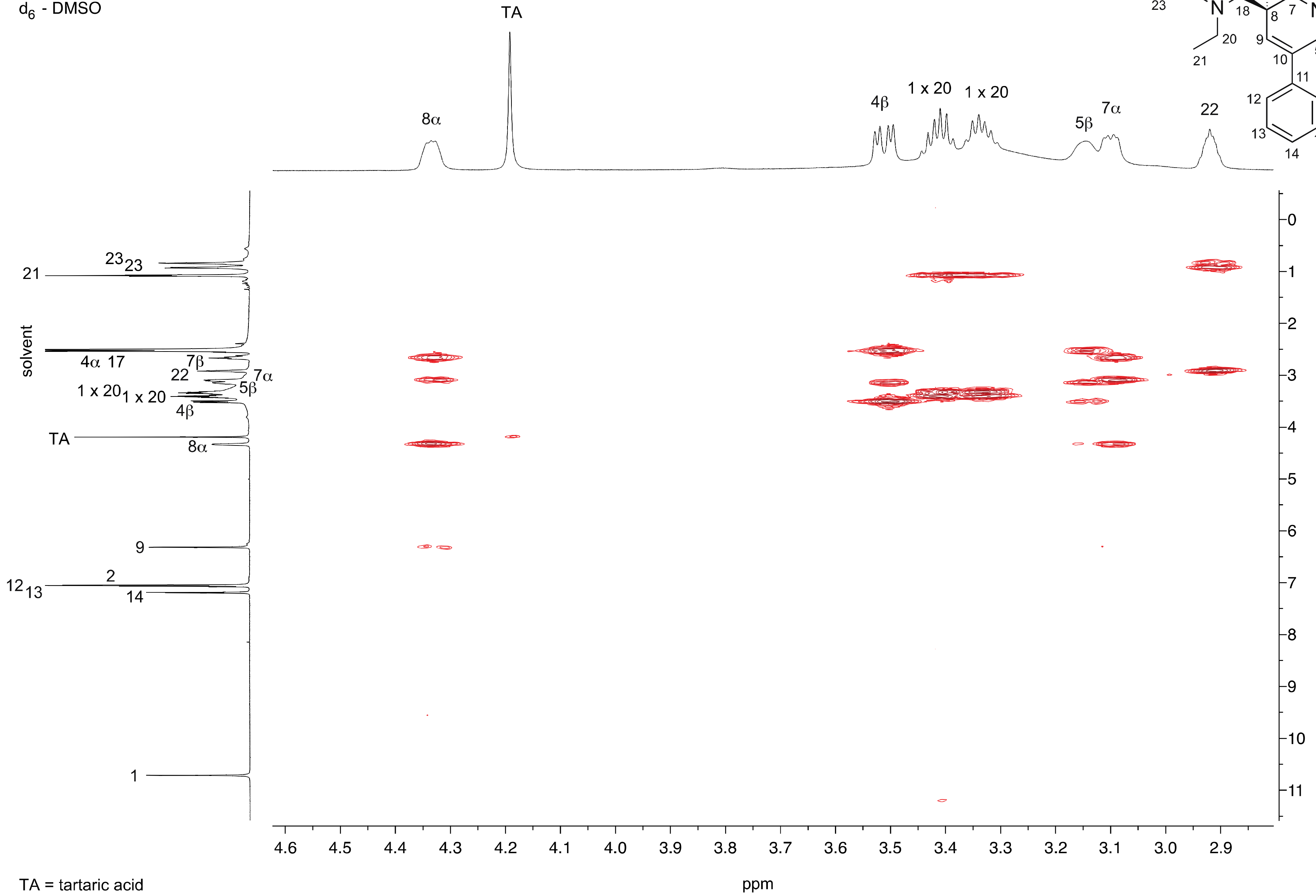

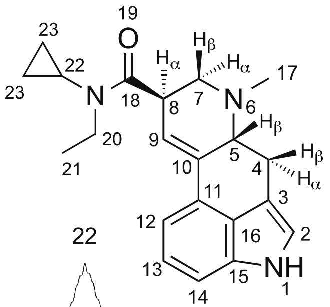

TA = tartaric acid

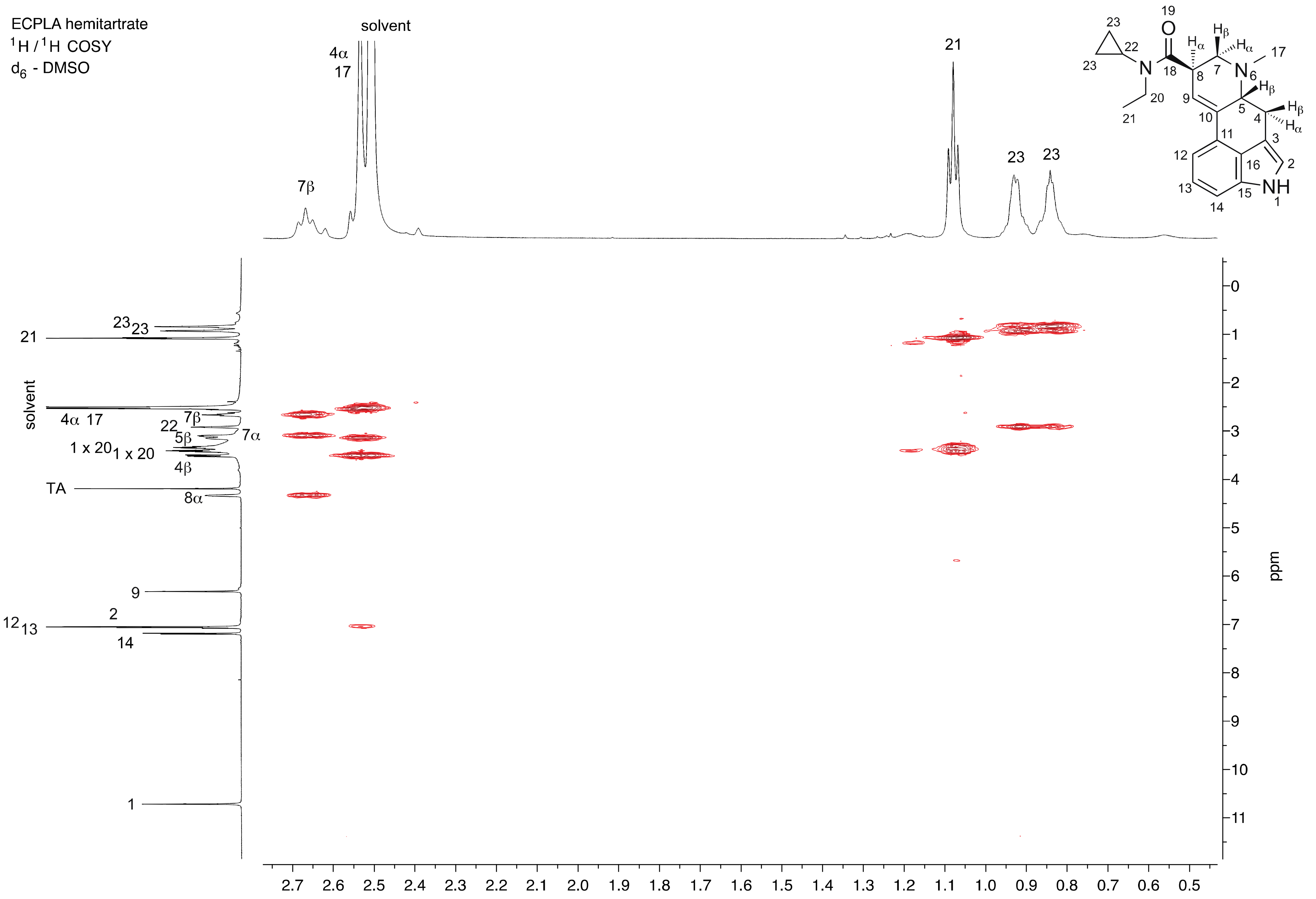

TA = tartaric acid

ECPLA hemitartrate
^{13}C-NMR (150 MHz)
d_6-DMSO

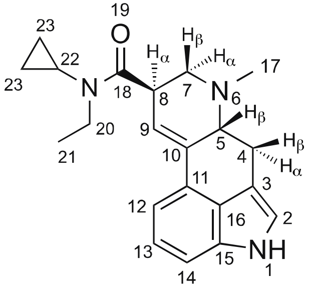

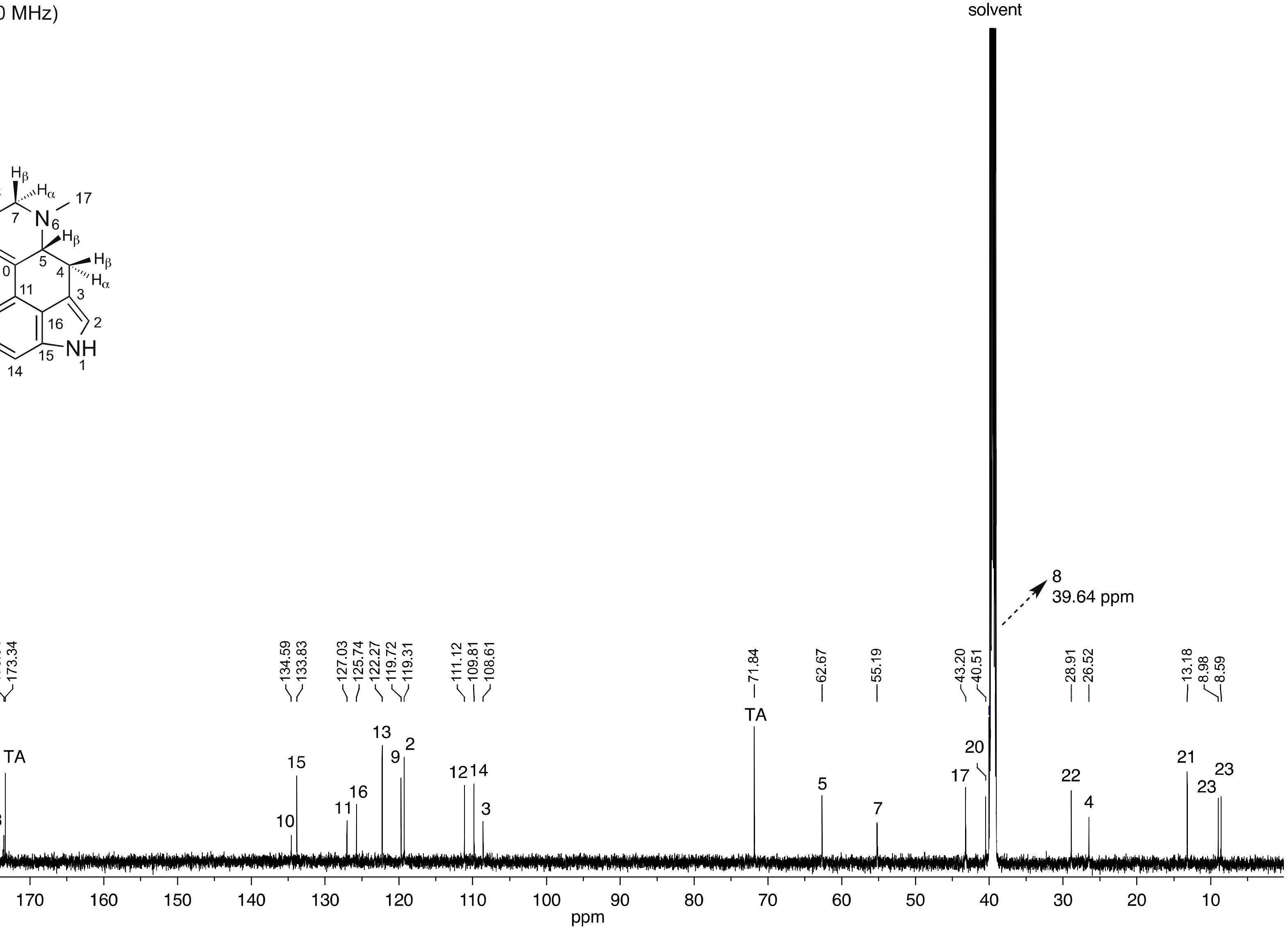

TA = tartaric acid

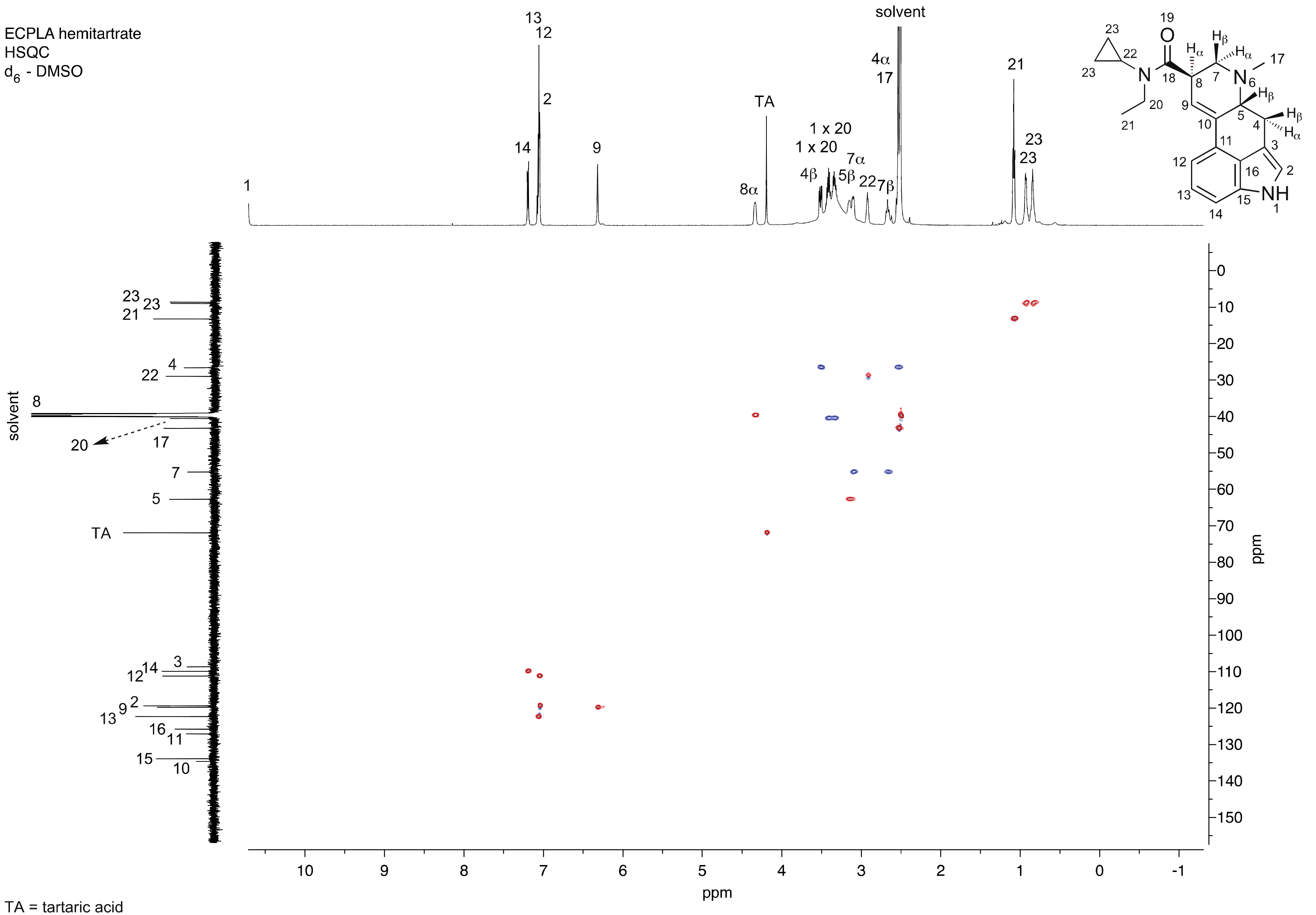

TA = tartaric acid

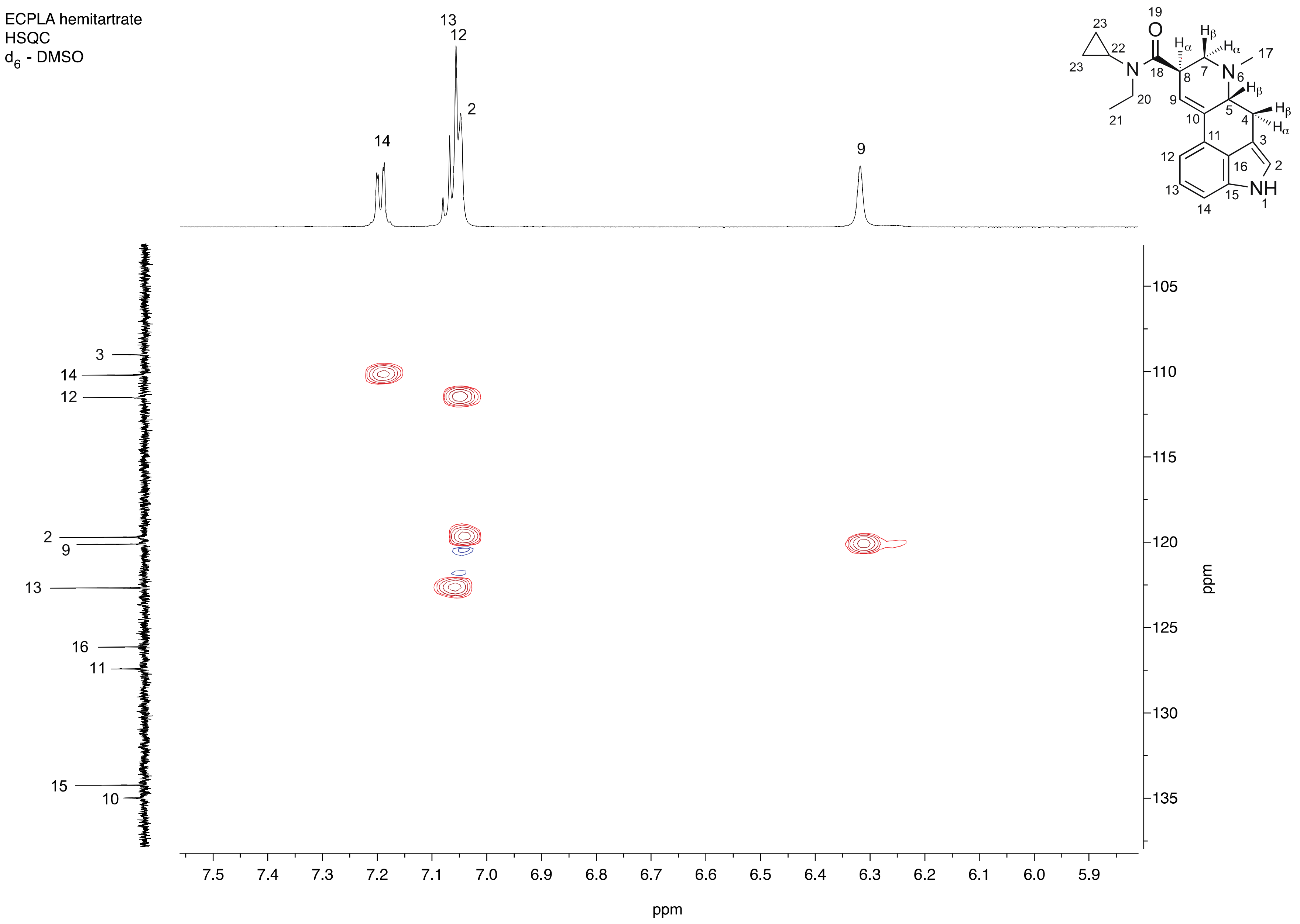
ECPLA hemitartrate
HSQC
d6 - DMSO
13
12
2
14
9
3
14
12
2
9
13
16
11
15
10
105
110
115
120
125
130
135
ppm
7.5
7.4
7.3
7.2
7.1
7.0
6.9
6.8
6.7
6.6
6.5
6.4
6.3
6.2
6.1
6.0
5.9
ppm

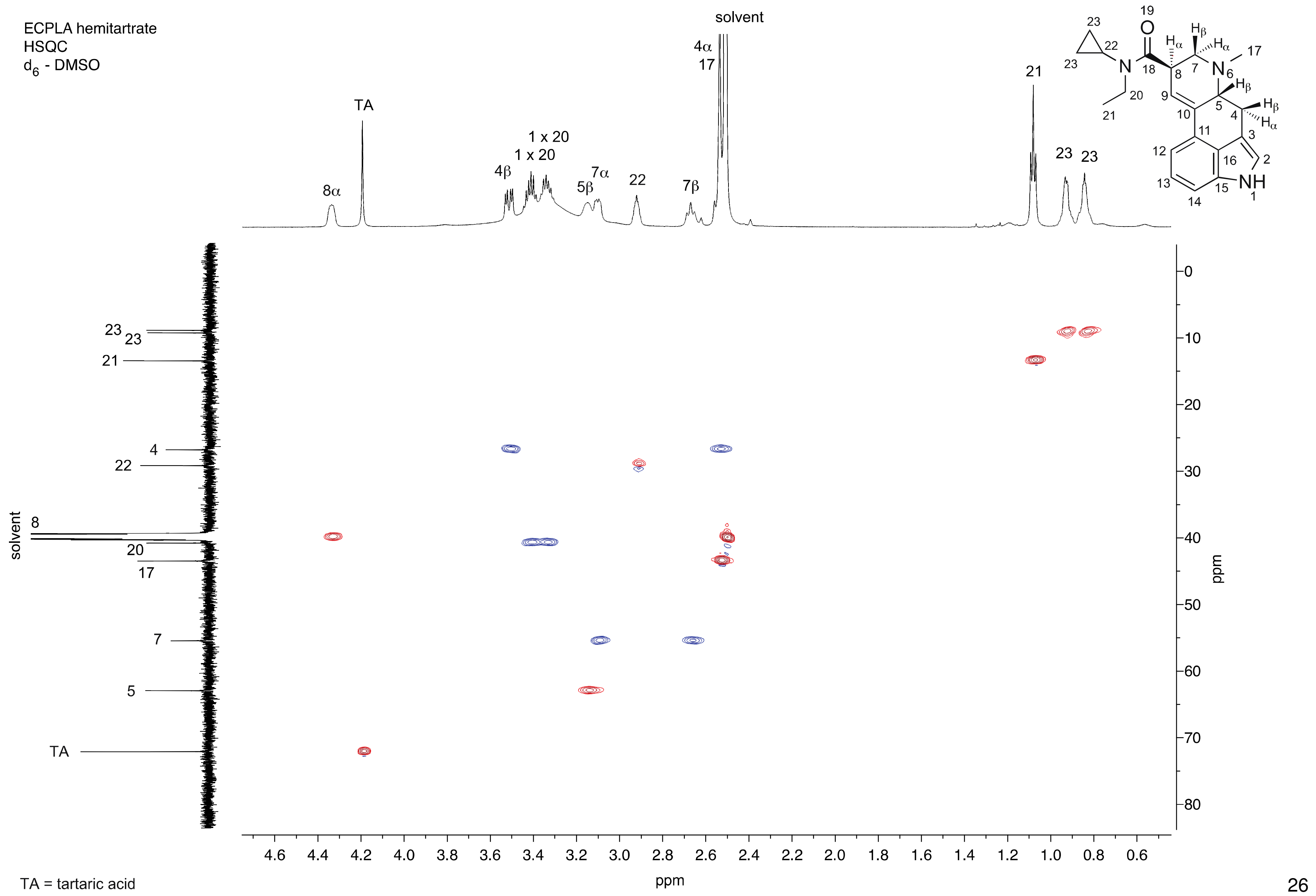
ECPLA hemitartrate
HSQC
d6 - DMSO
solvent
TA
8α
4β
1 x 20
1 x 20
5β
7α
22
7β
4α
17
21
23
23
ppm
solvent
TA
5
7
17
20
8
22
4
21
23
23
TA = tartaric acid

ECPLA hemitartrate
HMBC
d_6 - DMSO

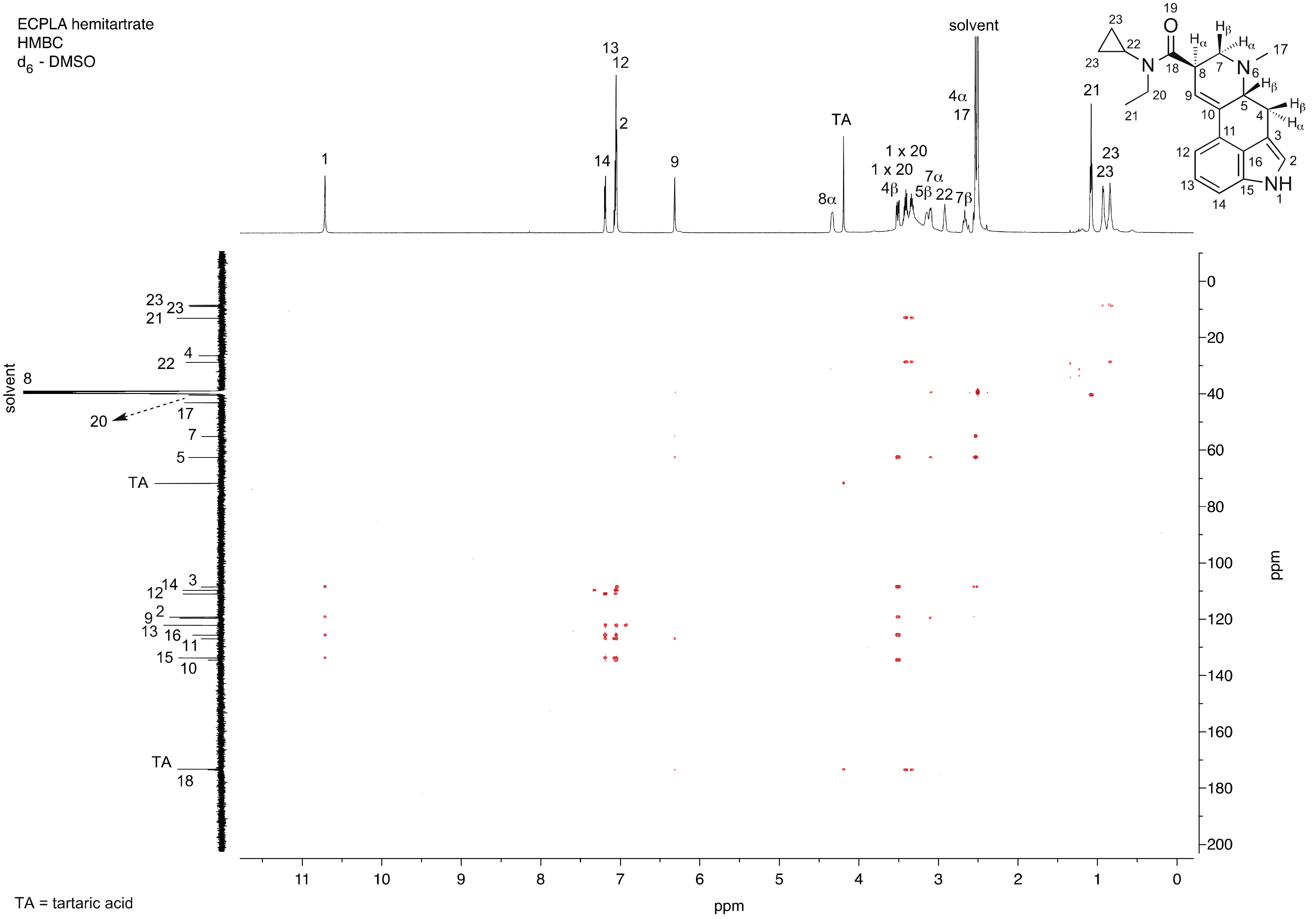

TA = tartaric acid

ECPLA hemitartrate
HMBC
d_6 - DMSO

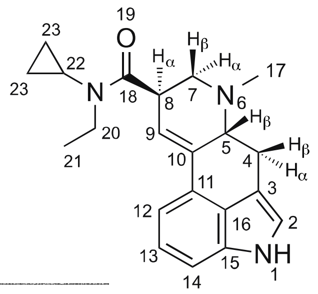

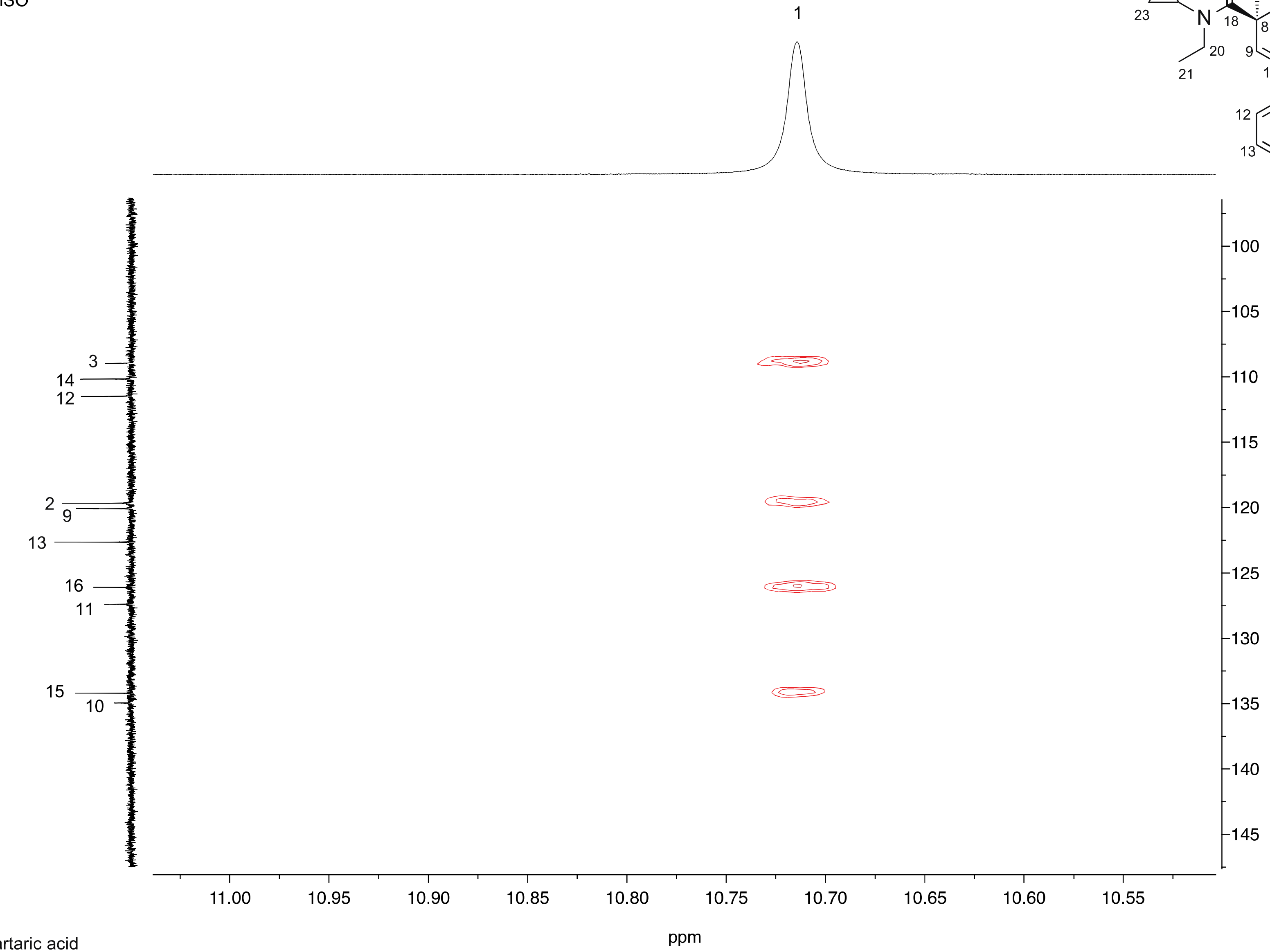

TA = tartaric acid

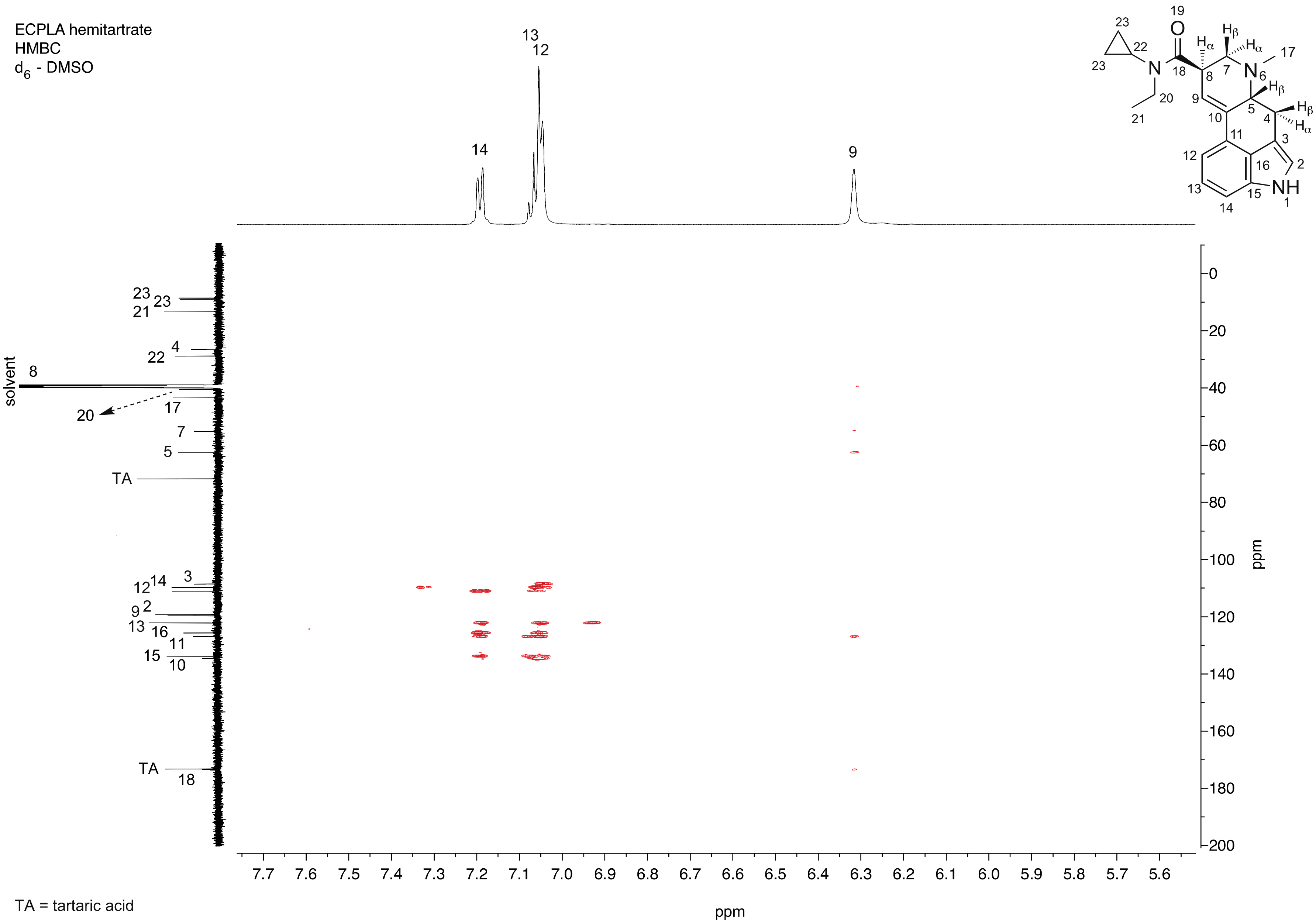
ECPLA hemitartrate
HMBC
d6 - DMSO
13
12
14
9
23
22
19
O
18
N
20
21
8
7
N
6
17
5
4
3
2
16
NH
1
15
14
13
12
11
10
9
solvent
TA
TA = tartaric acid
ppm
ppm

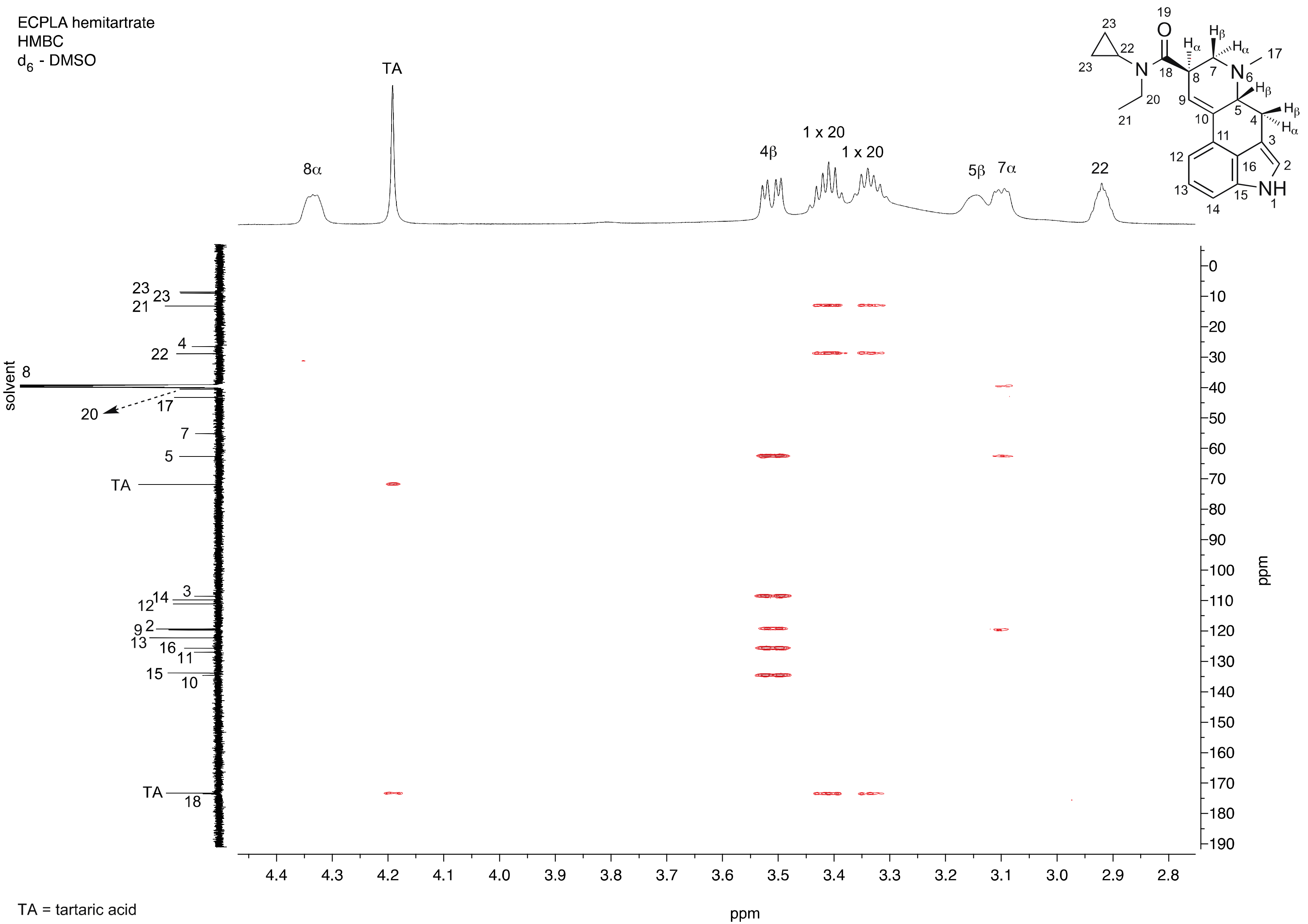

TA = tartaric acid

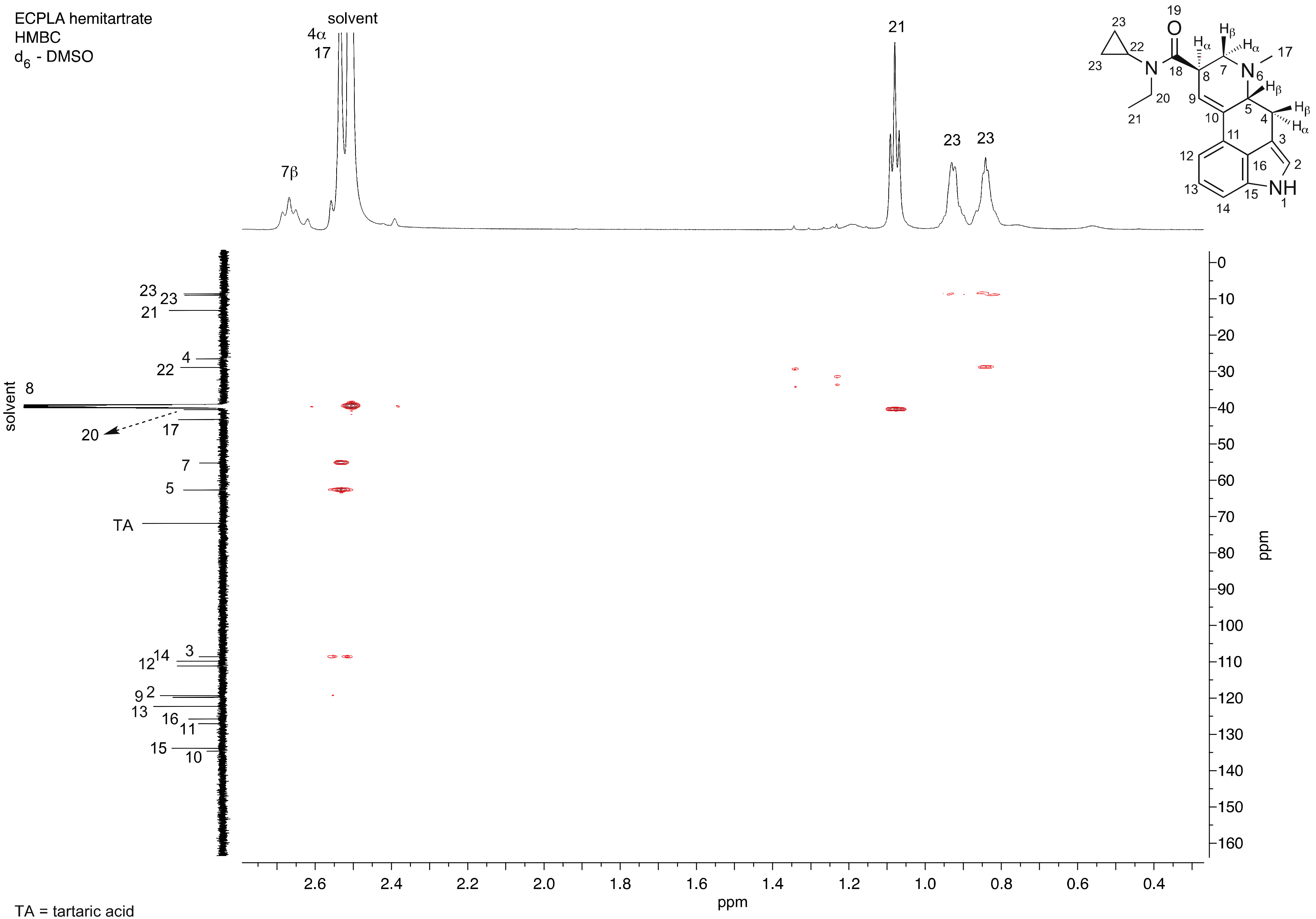

TA = tartaric acid

ECPLA hemitartrate
^{1}H NMR / 600 MHz
CD_3OD

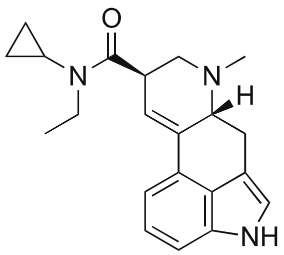

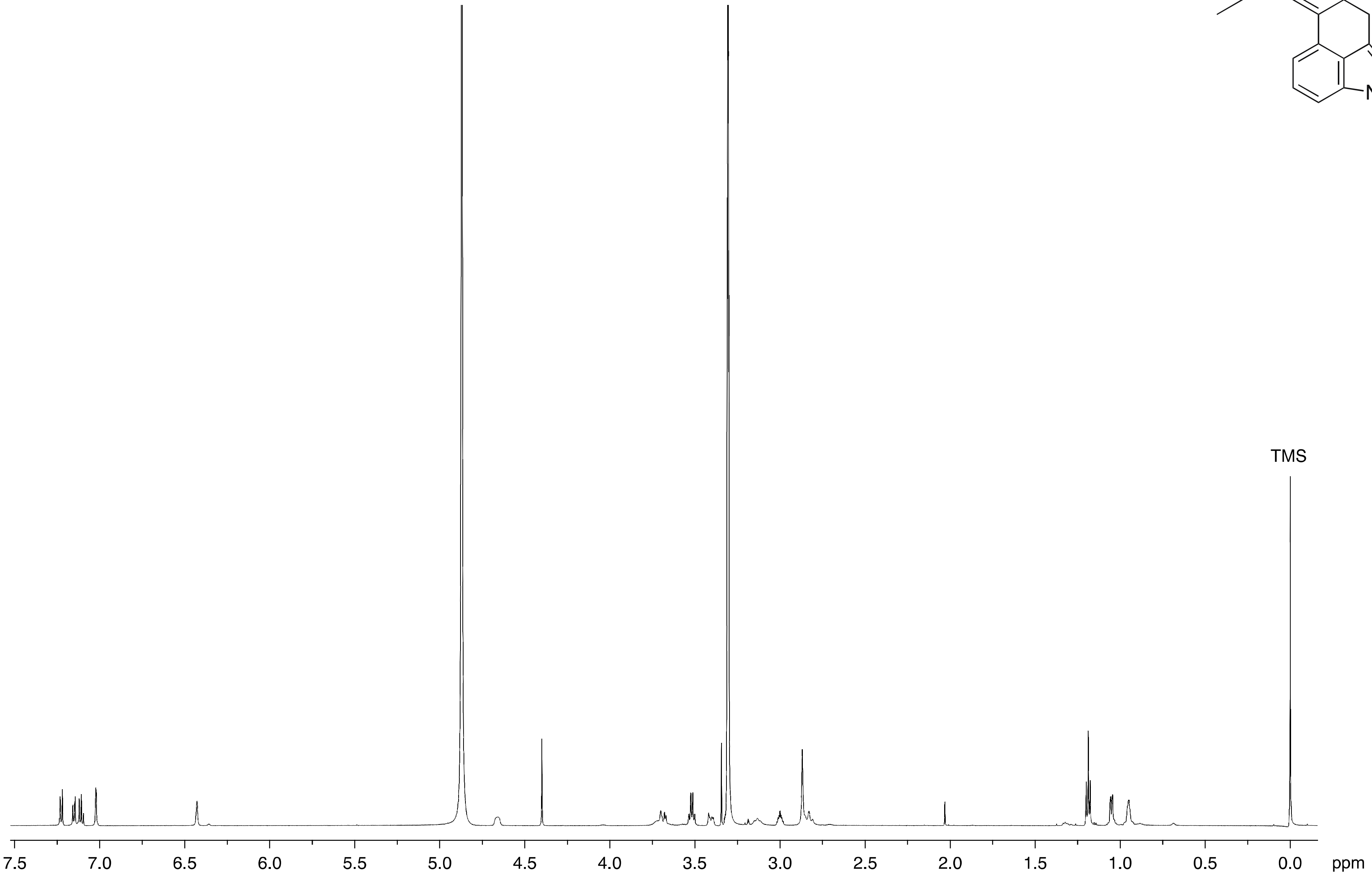

ECPLA hemitartrate
^{1}H NMR / 600 MHz
CD_3OD

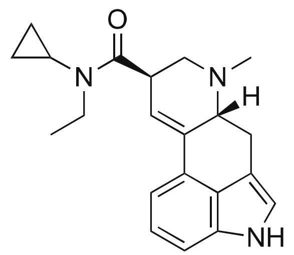

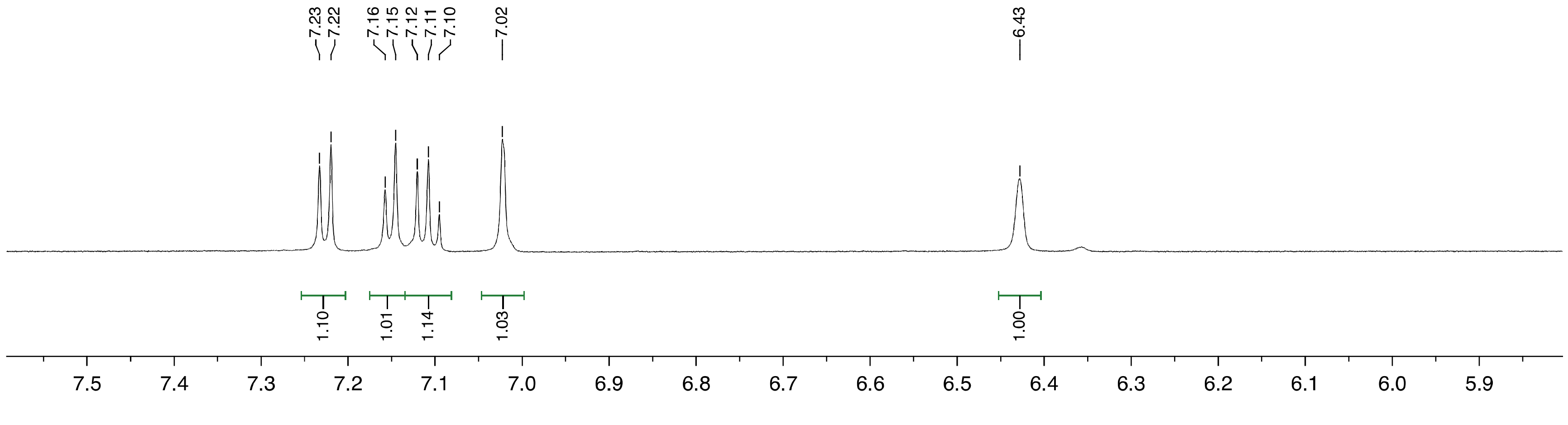

ECPLA hemitartrate
^1H NMR / 600 MHz
CD_3OD

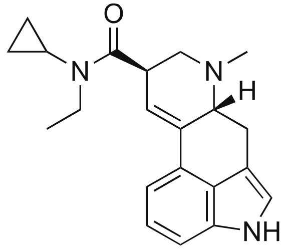

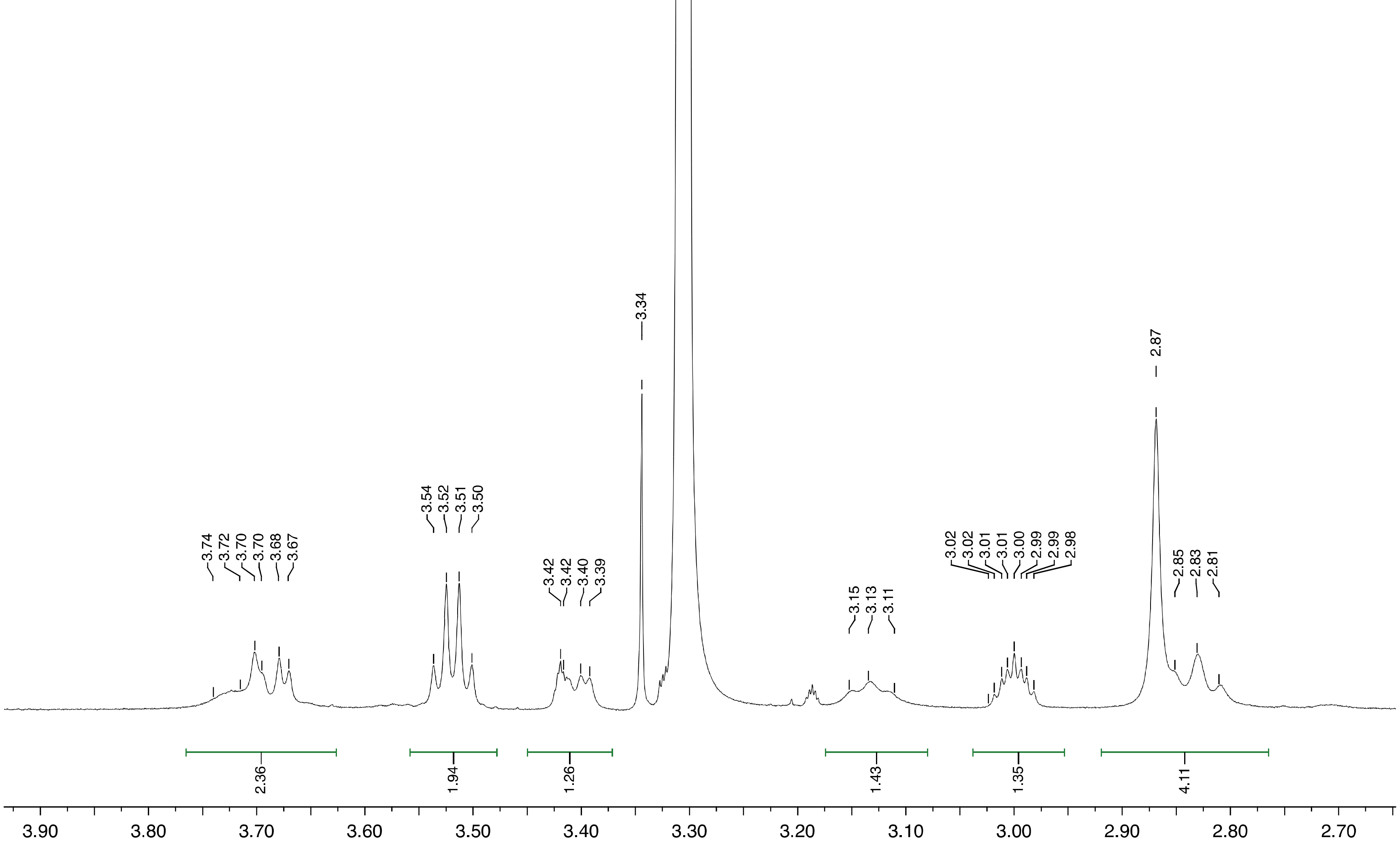

ECPLA hemitartrate
^{1}H NMR / 600 MHz
CD_3OD

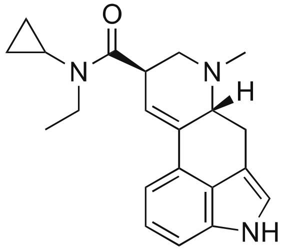

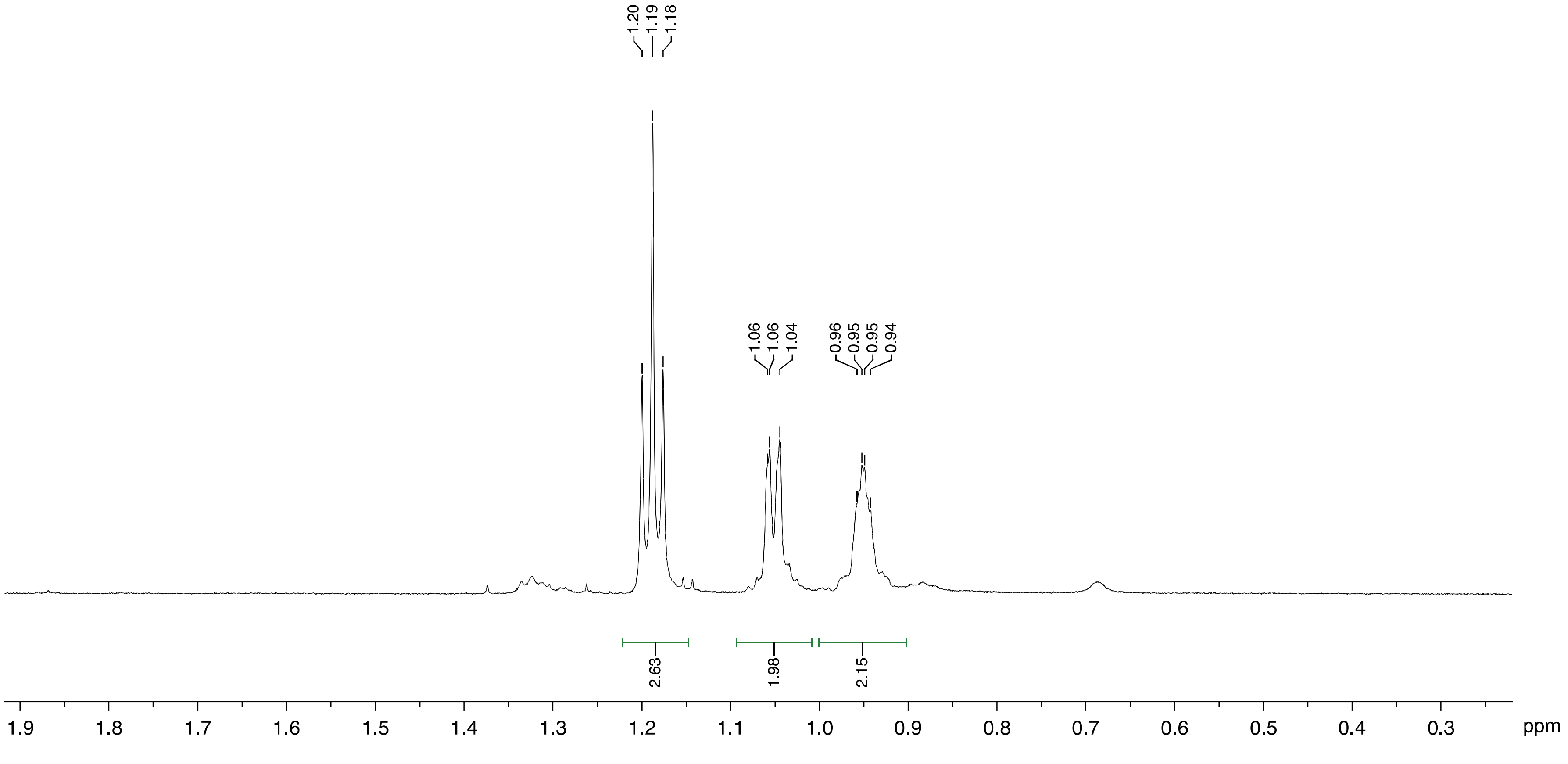

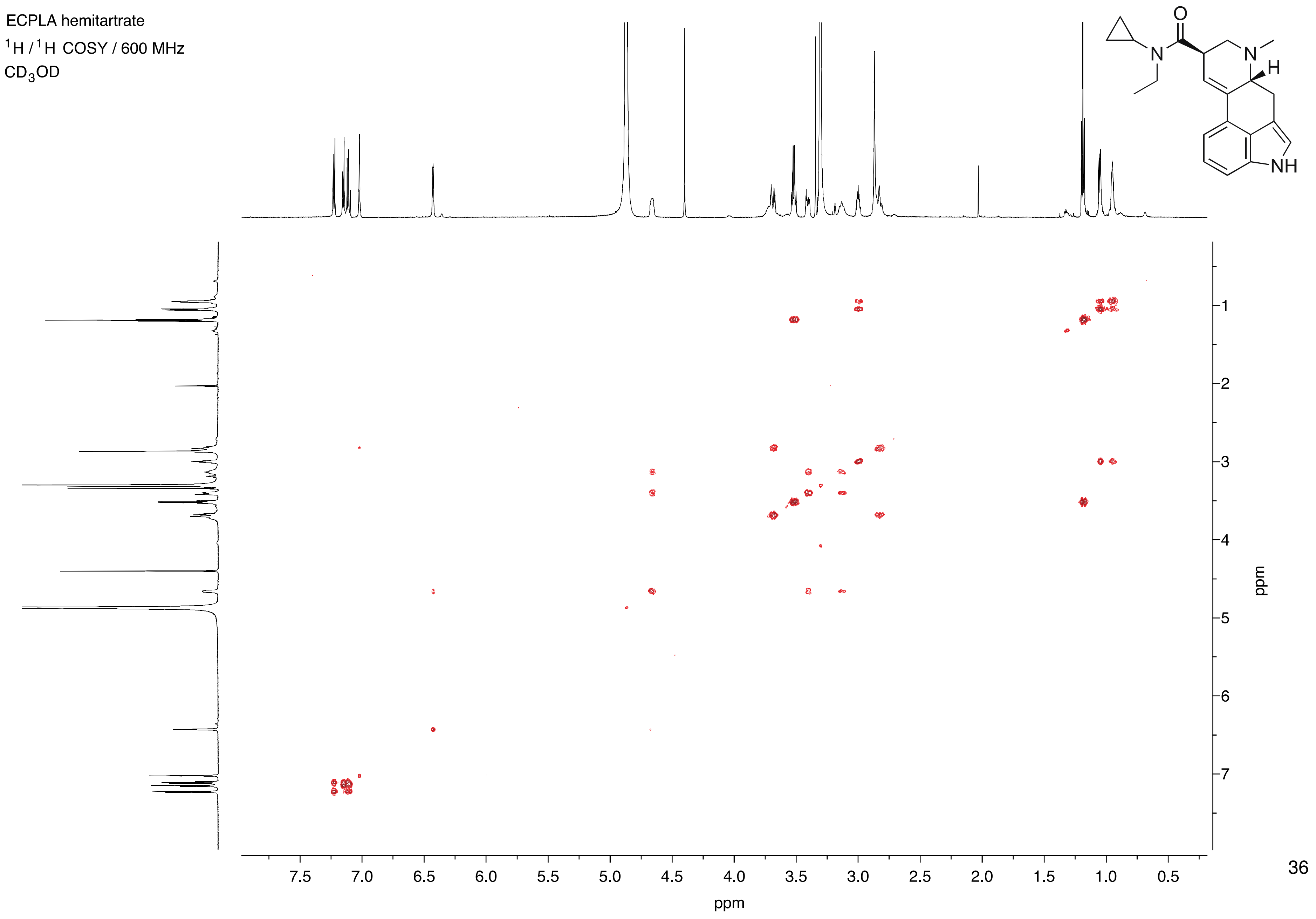
ECPLA hemitartrate
1H / 1H COSY / 600 MHz
CD3OD
O
N
N
H
NH
7.5
7.0
6.5
6.0
5.5
5.0
4.5
4.0
3.5
3.0
2.5
2.0
1.5
1.0
0.5
ppm
1
2
3
4
5
6
7
ppm

ECPLA hemitartrate

^{1}H / ^{1}H COSY / 600 MHz

CD_3OD

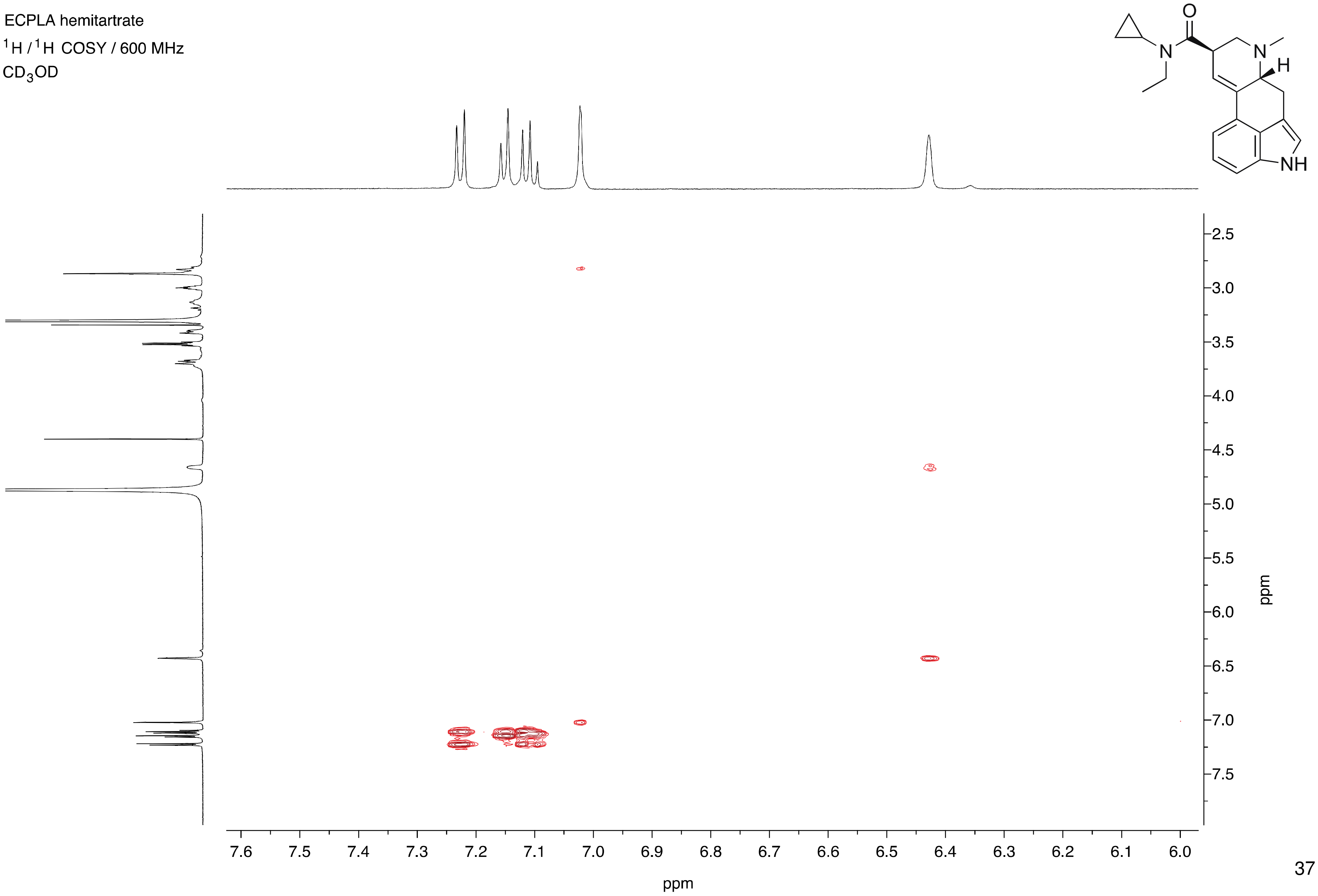

ECPLA hemitartrate
^{1}H / ^{1}H COSY / 600 MHz
CD_3OD

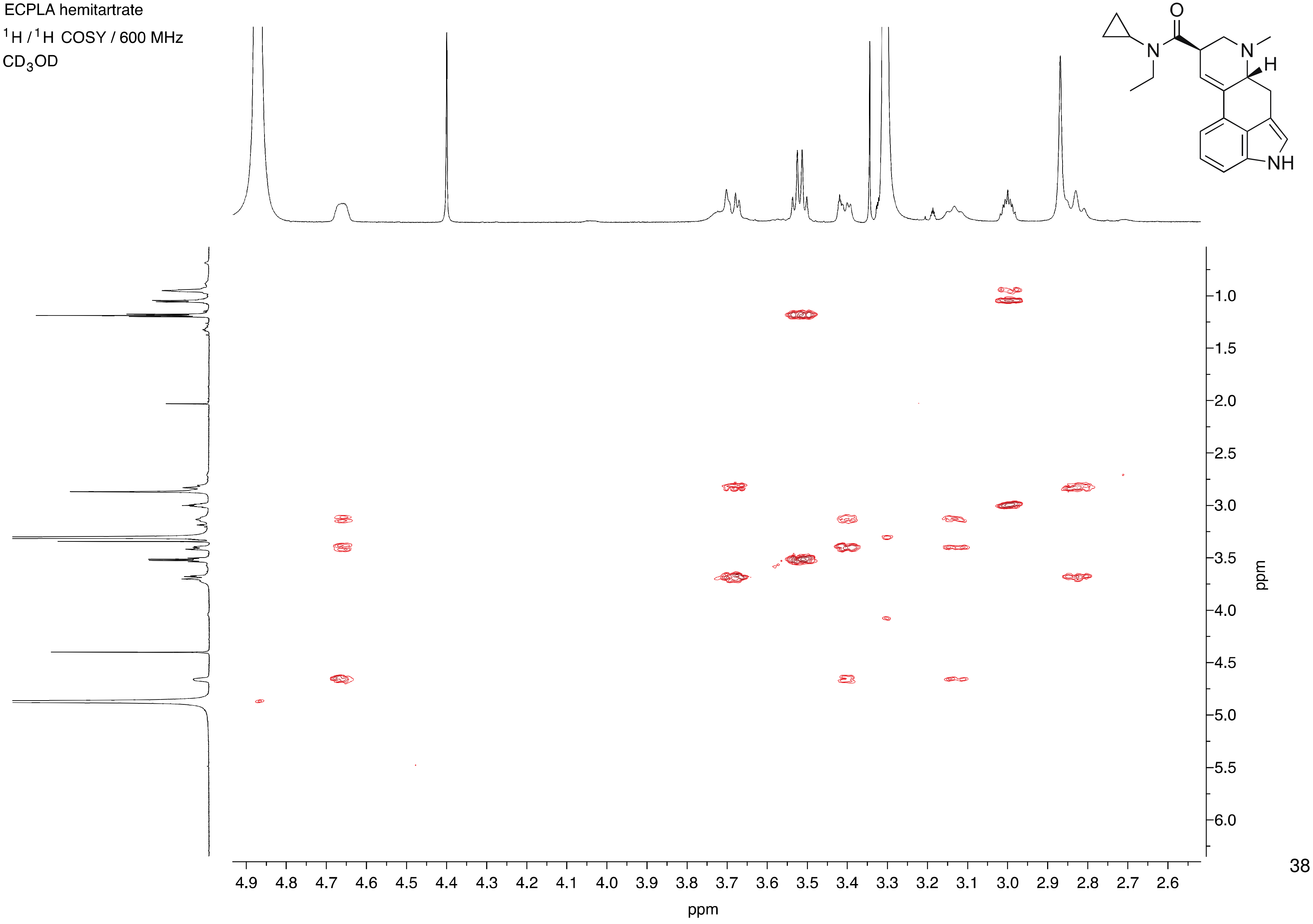

ECPLA hemitartrate
^1H / ^1H COSY / 600 MHz
CD_3OD

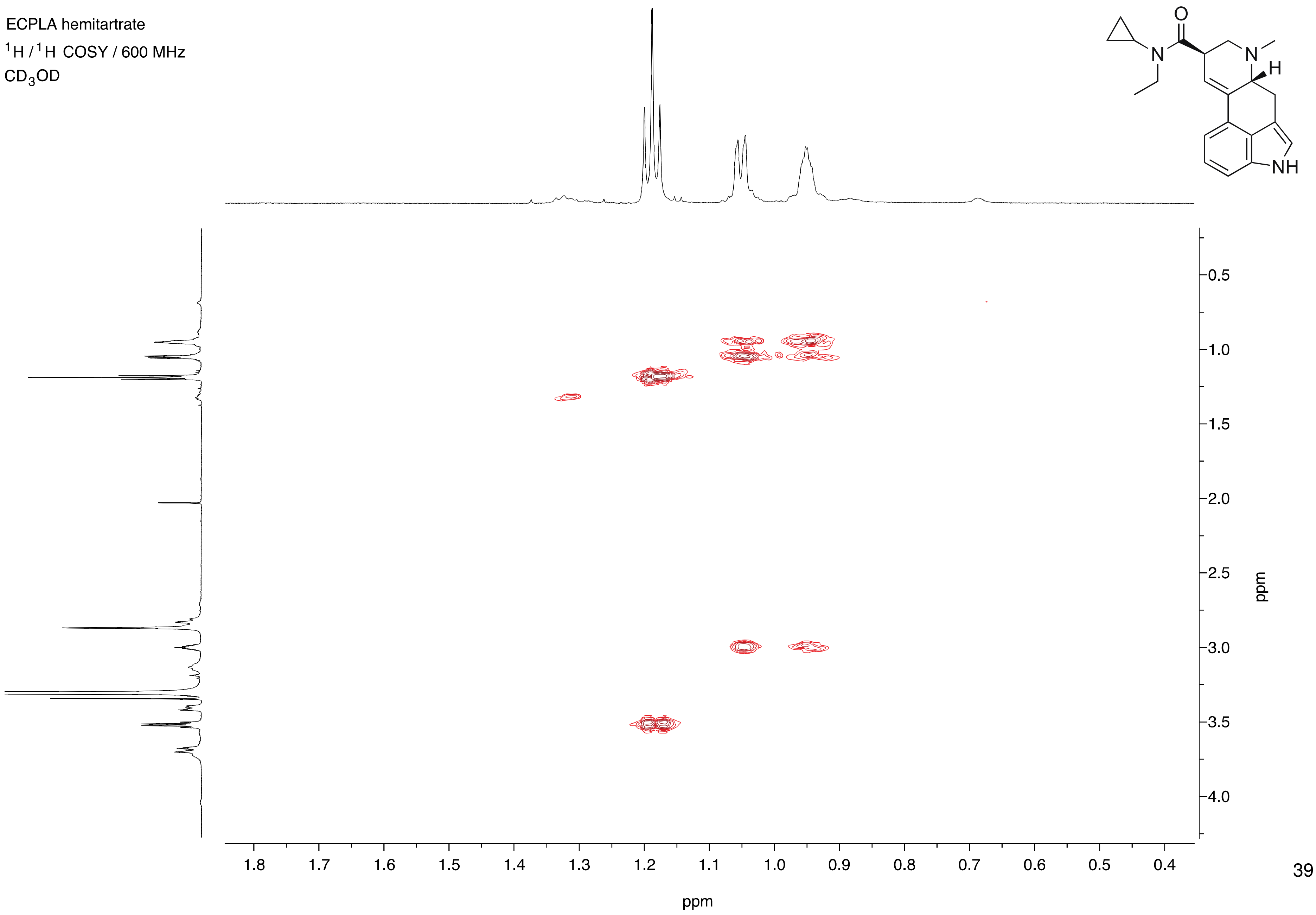

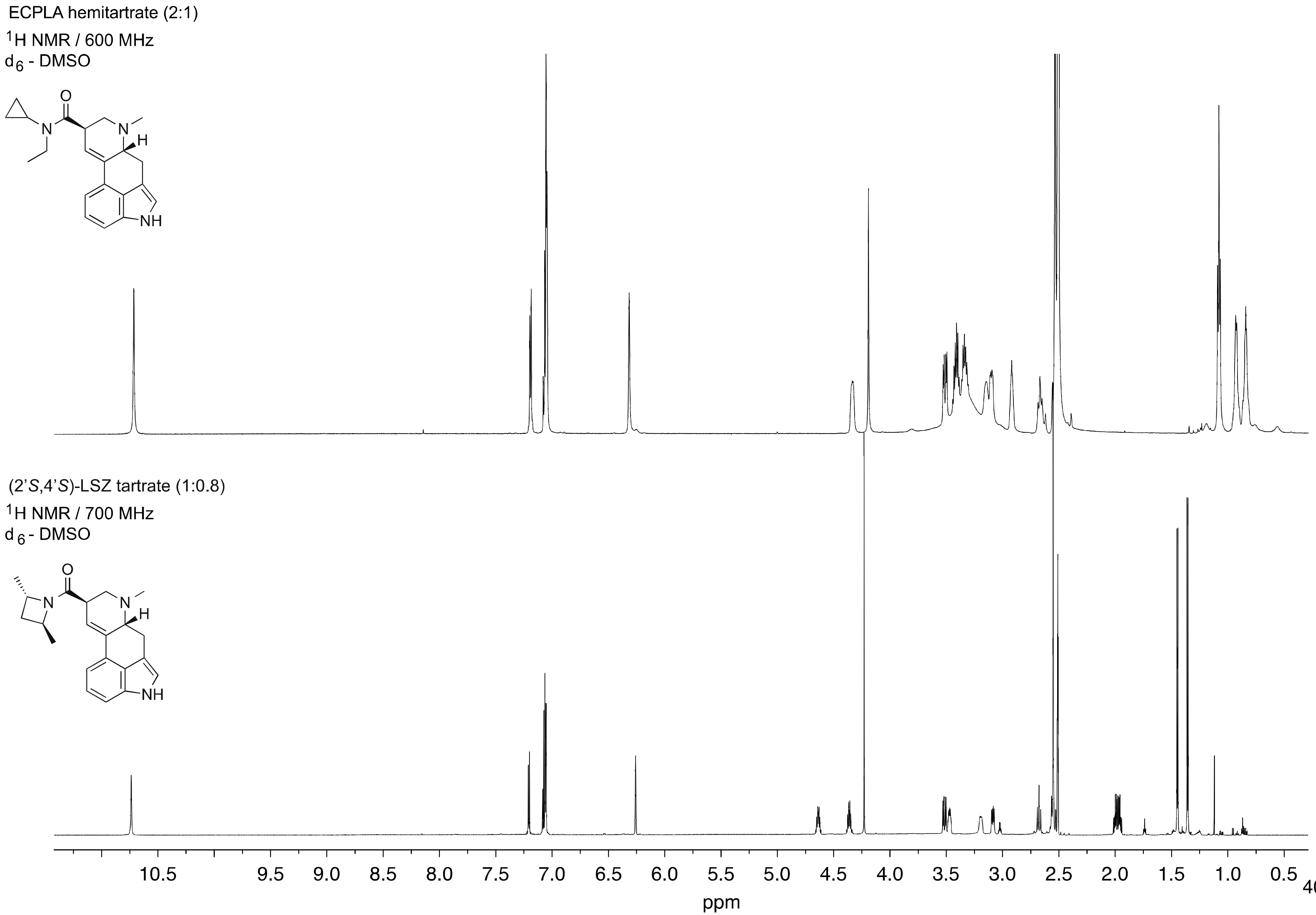

LSZ: Brandt et al. Drug Test Anal. 2017;9(1):38-50

ECPLA hemitartrate (2:1)
^1H NMR / 600 MHz
d_6 - DMSO

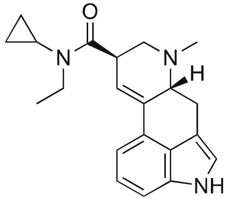

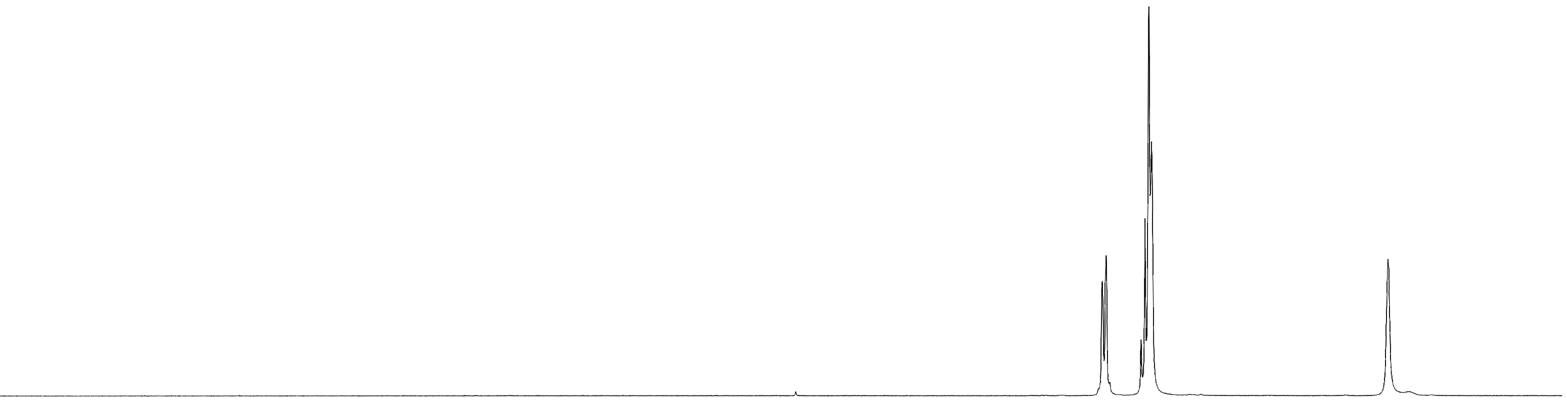

(2'*S*,4'*S*)-LSZ tartrate (1:0.8)
^1H NMR / 700 MHz
d_6 - DMSO

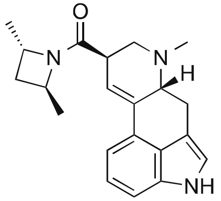

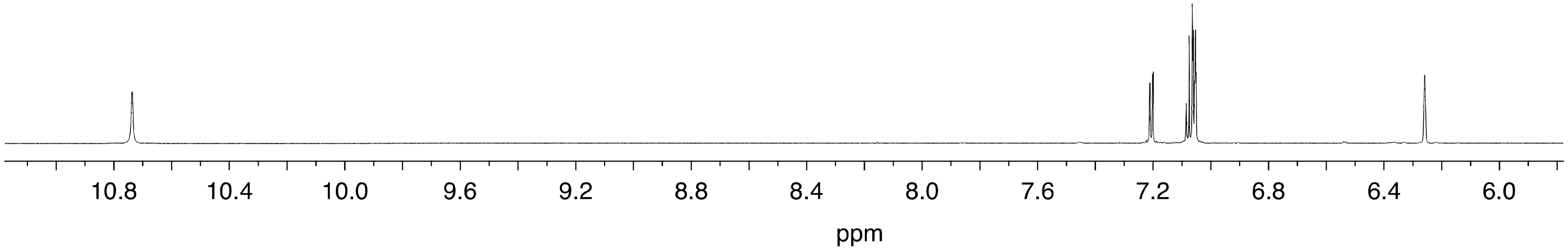

LSZ: Brandt et al. Drug Test Anal. 2017;9(1):38-50

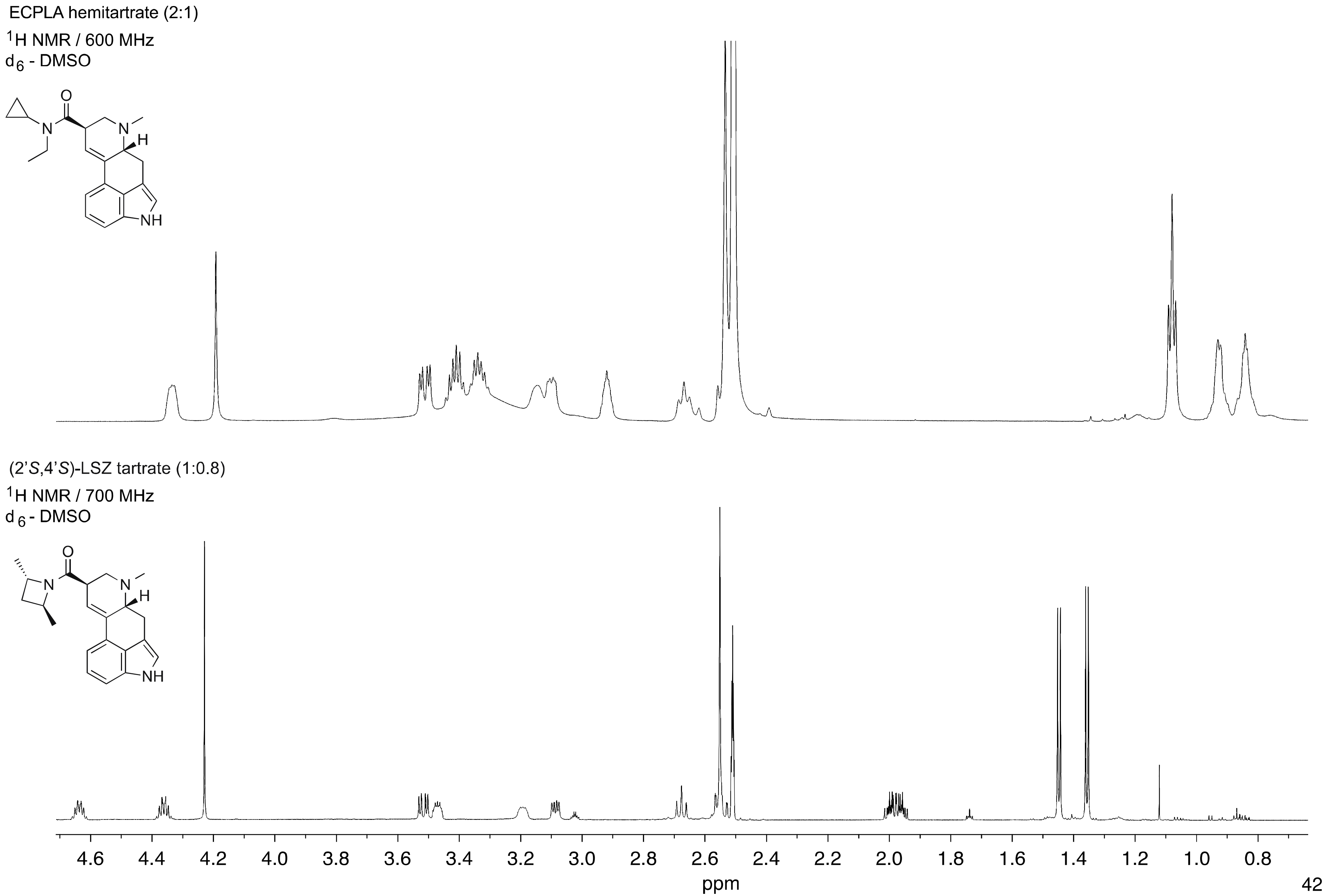

LSZ: Brandt et al. Drug Test Anal. 2017;9(1):38-50

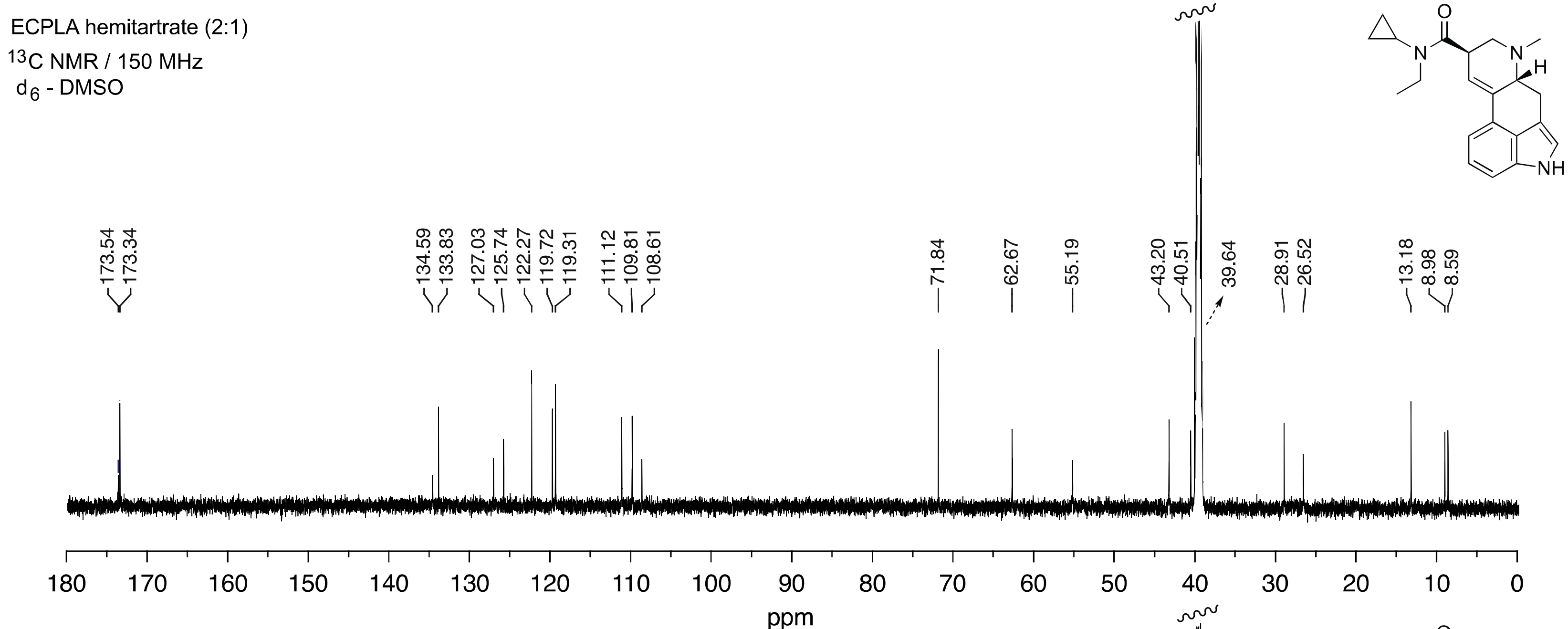

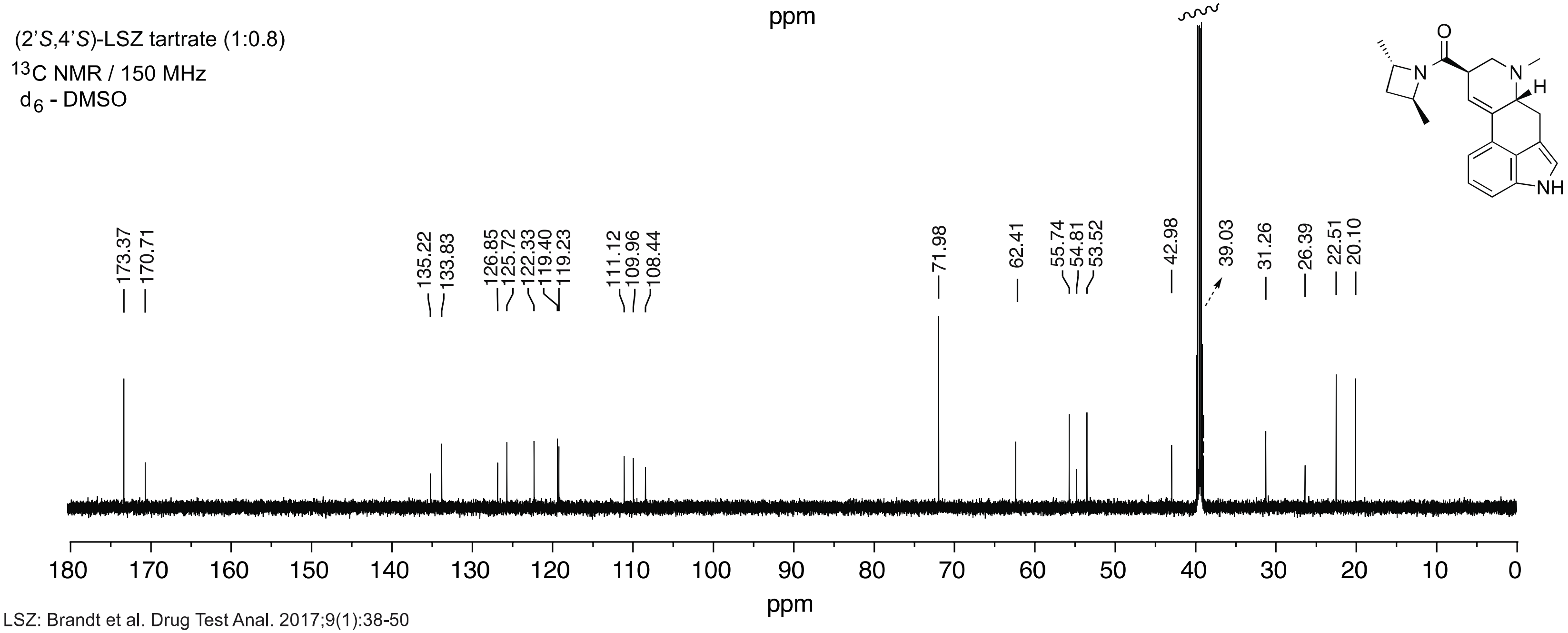

LSZ: Brandt et al. Drug Test Anal. 2017;9(1):38-50

Table S1. Three examples also reflecting the $C_{21}H_{25}N_3O$ formula (Mw 335.45 g/mol)		
**Compound**	**Chemistry**	**Pharmacology**
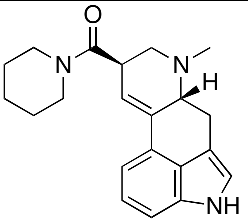<br>LSD-Pip;<br>LA-Pip;<br>Lysergic acid piperidide;<br>*N*-Piperidinyllysergamide	1-5	6-10
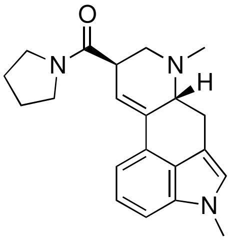<br>MPD-75;<br>1-Methyl-*N*-pyrrolidyllysergamide	11-14	15-20
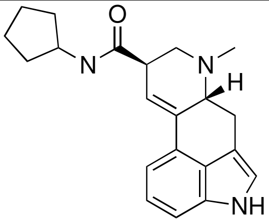<br>Cepentyl;<br>C_5AL;<br>*N*-Cyclopentyllysergamide	4,21-25	21,22,26-29

References

1. Stoll A, Hofmann A. Amide der stereoisomeren Lysergsäuren und Dihydro-lysergsäuren. 38. Mitteilung über Mutterkornalkaloide. *Helv Chim Acta.* 1955;38(2):421-433.
2. Votava Z. Pharmacologie des cycloalkyamides de l'acide d-lysergique. *J Physiol.* 1957;49:417-419.
3. Sandoz Ltd, Basle, Switzerland. Amide-like derivatives of lysergic and isolysergic acids. GB785173A; 1957.
4. Pioch RP. Eli Lilly and Company, Indianapolis, Indiana, USA. Lysergic acid amides. US2997470; 1961.
5. Nakahara Y, Niwaguchi T. Lysergic acid diethylamide and related compounds. III. Improvement of amidation of lysergic acid. *Yakugaku Zasshi.* 1974;94(3):407-412.

6. Cerletti A, Doepfner W. Comparative study on the serotonin antagonism of amide derivatives of lysergic acid and of ergot alkaloids. *J Pharmacol Exp Ther.* 1958;122:124-136.
7. Braden MR. Towards a biophysical understanding of hallucinogen action. Ph. D. Thesis, Purdue University, West Lafayette, IN, USA. 2007.
8. Parrish JC. Toward a molecular understanding of hallucinogen action. Ph. D. Thesis, Purdue University, West Lafayette, IN, USA. 2006.
9. Nichols DE. Structure-activity relationships of serotonin 5-HT$_{2A}$ agonists. *WIREs Membr Transp Signal.* 2012;1(5):559-579.
10. Nichols DE. Chemistry and structure-activity relationships of psychedelics. *Curr Top Behav Neurosci.* 2017;36:1-43.
11. Troxler F, Hofmann A. Substitutionen am Ringsystem der Lysergsäure II. Alkylierung. 44. Mitteilung über Mutterkornalkaloide. *Helv Chim Acta.* 1957;40(6):1721-1732.
12. Sandoz Ltd, Basle, Switzerland. New derivatives of the lysergic acid series and process for their manufacture. GB811964A; 1959.
13. Sandoz SA, Basle, Switzerland. Nouveaux amides héteérocyclique et leur préparation. BE613232A; 1962.
14. Hoffman A, Troxler F, Ott H. Sandoz AG, Basle, Switzerland. Verfahren zur Herstellung von heterozyklischen Verbindungen. CH401075A; 1966.
15. Shulgin AT. Psychotomimetic agents. In: Gordon M, ed. *Psychopharmacological Agents.* Vol 4. New York: Academic Press; 1976:59-146.
16. Shulgin AT. Hallucinogens. In: Wolf ME, ed. *Burger's Medicinal Chemistry, Part III, 4th ed*. New York: John Wiley and Sons, Inc.; 1981:1109-1137.
17. Shulgin AT. Chemistry of psychotomimetics. In: Hoffmeister F, Stille G, eds. *Handbook of Experimental Pharmacology. Psychotropic Agents, Part III: Alcohol and Psychotomimetics, Psychotropic Effects of Central Acting Drugs.* Vol 55(3)1982:3-29.
18. Jacob III P, Shulgin AT. Structure-activity relationships of the classic hallucinogens and their analogs. In: Lin GC, Glennon RA, eds. *Hallucinogens: An update. NIDA Research Monograph 146*. Wahington, DC, USA: U.S. Department of Health and Human Services, National Institute of Health, U.S. Government Printing Office; 1994:74-91.
19. Shulgin AT. Basic pharmacology and effects. In: Laing R, Siegel JA, eds. *Hallucinogens. A Forensic Drug Handbook*. London: Academic Press; 2003:67-137.
20. Hofmann A. *Die Mutterkornalkaloide: Vom Mutterkorn zum LSD - Die Chemie der Mutterkornalkaloide.* Solothurn, Switzerland: Nachtschatten Verlag; 2000.
21. Semonsky M, Zikan V, Votava Z. Námelové alkaloidy VIII. Parciální synthesa několika cykloalkylamidů kyselin d-*iso*-lysergové a d-lysergové. *Chem Listy Vedu Prum.* 1957;51:592-596.
22. Votava Z, Podvalová I, Semonský M. Studies on the pharmacology of d-lysergic acid cycloalkylamides. *Arch Int Pharmacodyn Ther.* 1958;115:114-130.
23. Semonsky M, Zikan V. Prague, Czechoslovakia. Cycloalkylamidesof *d*-ysergic acid and method of producing the same. GB816273A; 1959.
24. Macek K, Vaněček S. Mutterkornalkaloide XXIV. Papierchromatographie der Lysergsäurecycloalkylamide und N-Methylergolinyl-N'-cycloalkylharnstoffe. *Pharmazie.* 1962;17:442-444.
25. Hofmann A, Troxler F. Sandoz AG, Basle, Switzerland. Verfahren zur Herstellung von am Indolstickstoff substituierten Derivaten der Lysergsäure-Reihe. CH386441A; 1965.
26. Votava Z, Podvalová I, Semonský M. Oxytocic effect of some d-lysergic acid cycloalkyl amides. *Nature.* 1957;179:474-475.
27. Hladovec J, Votava Z. The effect of ergot alkaloids, their partial synthetic derivatives and serotonin on blood clotting. *Physiol Bohem.* 1958;7:553-558.

28. Wiegershausen B. Some pharmacological effects of staphylolysin on the isolated rat uterus. *Nature.* 1959;184(4704):2017-2017.
29. Votava Z, Lamplova I. Antiserotonin effect of derivatives of D-lysergic acid (cycloalkylamides, lysergic acid diethylamide, ergometrine) and chlorpromazine in rats. *Act Nerv Super.* 1959;1:269-275.
